# Ethnobotanical characterization of medicinal plants used in Kisantu and Mbanza-Ngungu territories, Kongo-Central Province in DR Congo

**DOI:** 10.1186/s13002-020-00428-7

**Published:** 2021-01-23

**Authors:** Kibungu Kembelo Pathy, Nzuki Bakwaye Flavien, Belesi Katula Honoré, Wouter Vanhove, Van Damme Patrick

**Affiliations:** 1 Department of Environmental Sciences, Kinshasa University (UNIKIN), Kinshasa XI, BP 127 Democratic Republic of the Congo; 2 Laboratory of Tropical and Subtropical Agriculture and Ethnobotany, Ghent University, Coupure Links 653, B-9000 Ghent, Belgium; 3 Faculty of Tropical AgriSciences, Czech University of Life Sciences Prague, Kamycka 129, Prague 6 – Suchdol, 165 21, Prague, Czech Republic

**Keywords:** Phytopharmacopeia, Medicinally important species, Use-value consensus, Informant knowledge, Cultural similarity, Kisantu, Mbanza-Ngungu

## Abstract

**Background:**

The phytotherapeutic knowledge of the Kongo people in the territories of Kisantu and Mbanza-Ngungu in Kongo-Central Province (DR Congo) is rapidly eroding. To document the remaining knowledge, we conducted an ethnobotanical survey on the most important medicinal plant species and diseases treated with them, as well as plants with therapeutic potential. We also checked for the cultural similarity in medicinal plant knowledge between the two territories and how knowledge about Kongo medicinal plants differs between different social groups.

**Methods:**

From June 2017 until February 2018 and from February 2019 until April 2019, we conducted a survey with 188 phytotherapists, selected using the snowball method and surveyed using semi-structured interviews. Voucher specimens were taken for identification. Ethnobotanical data were analyzed using medicinal use value (UVs), informant agreement ratio (IARs), informant consensus factor (ICF), and species therapeutic potential (STP). Rahman’s similarity index was used for ethno-cultural comparison of medicinal plant knowledge between the two communities. Medicinal knowledge between different social groups was analyzed using non-parametric tests and Poisson regression.

**Results:**

A total of 231 plants (i.e., 227 botanical species, representing 192 genera and 79 families) were reportedly used to treat 103 diseases. Most abundant taxa were reported for the Fabaceae family (including 11.9% of species and 10.9% of genera). Most reported species (45.0%) were from anthropized areas. Leaves (39.4%), herbs (37.1%), decoction (41.7%), and oral ingestion (72%) were the most frequently cited plant part, botanical form, preparation, and administration method, respectively. Four of all inventoried species showed high UV_S_ (> 0.05), whereas eight had an IAR of one. According to ICF, 31 diseases were mentioned. Highest ICF (≥ 0.4) was observed for hemorrhoids (0.44), amoebiasis (0.43), and itchy rash (0.42). Fifty-four plant species were identified as likely possessing an interesting therapeutic potential. Low ethno-cultural similarity in medicinal knowledge (RSI = 16.6%) was found between the two territories. Analysis of the Kongo medicinal plant knowledge showed that the mean number of reported species and diseases vary considerably depending on gender, type, and residence of therapists (*P* < 0.05).

**Conclusion:**

Results prove that the Kongo phytopharmacopeia makes use of interesting medicinal plant species that could be further studied for conservation and pharmacological applications.

**Supplementary Information:**

The online version contains supplementary material available at 10.1186/s13002-020-00428-7.

## Introduction

Since time immemorial, so-called medicinal plants have been used by all civilizations around the globe to treat various types of diseases [1, 2]. Today, medicinal plants still have the interest of modern medicine, the pharmaceutical sector in particular, in search of new drugs [3, 4]. It is estimated that around 80% of the global population still practices phytotherapy [5]. In Africa, as in most underdeveloped countries, extreme and widespread poverty limits people’s access to quality health care or modern medicine [6], forcing them to rely on herbal medicine [7].

Several studies have raised the issue of loss or risk of extinction of traditional knowledge and skills in medicinal plant use. They have identified the degradation, deterioration of the natural habitat, and the disinterest of young people in traditional culture due to westernization, acculturation, and education as the main causes of medicinal plant species’ disappearance [8, 9]. This problem also prevails in Kongo-Central Province (DR Congo) [10]. In 1983, Daeleman and Pauwels [11] reported the disappearance of *Erythrophleum suaveolens* (Guill. & Perr.) Brenan, a species formerly used in the practice of trial by poison by the Kongo people, which allowed to identify the culprit behind a disease of supposedly mystical origin. In herbal medicine, this species was and is still used against rheumatism and gynecological problems.

According to Makumbelo et al. [12] and Kibungu [13], some native, wild medicinal plant species in Kongo-Central Province have increasingly become rare. This is, e.g., the case for *Mondia whitei* (Hook. f.) Skeels, *Garcinia kola* Heckel, and *Dorstenia laurentii* De Wild., which are used against sexual impotence, abdominal pain, and intestinal amoebiasis, respectively. A recent medicinal plant vulnerability study in Mbanza-Ngungu [14] showed that *Lannea antiscorbutica* (Hiern) Engl*.*, *Mondia whitei* (Hook. f.) Skeels, *Monodora myristica* (Gaertn.) Dunal*, Pseudospondias microcarpa* (A. Rich.) Engl*.*, and *Annona senegalensis subsp. oulotricha* Le Thomas are—according to their vulnerability indices (Iv)—the most vulnerable species in Kongo herbal medicine.

Medicinal plant use and the accumulated knowledge of traditional phytotherapeutic practices are a rich cultural heritage and form an integral part of local culture and tradition, and should be safeguarded to ensure their continued use [15].

In Kisantu and Mbanza-Ngungu territories, ancestral knowledge and skills regarding medicinal plants are orally transmitted, making them vulnerable to erosion and extinction. Medicinal plant studies and documentation in the area can help to save and conserve them [13].

Our ethnobotanical research is based on the assumption that the Kongo phytomedicinal knowledge contains much interesting information about important local medicinal plant species with effective therapeutic potential. According to Nzuki [10], the most important medicinal plants, are those distinguished by their medicinal use-value (UVs) or informant agreement ratio (IAR). They are to be prioritized for both cultivation and conservation to prevent their disappearance.

Heinrich et al. [16] and Lautenschläger et al. [17] suggested that the informant consensus factor (ICF) is a good indicator for selecting plant species best adapted to pharmacological needs and to be subjected to phytochemical analysis.

The objectives of our study are to (1) identify the most important medicinal plants and their uses in Kongo phytotherapy, (2) asses the ethno-cultural similarity of medicinal plant use knowledge between the two territories and compare medicinal plant knowledge (number of species cited and number of diseases treated) between different social groups distinguished according to respondents’ gender, age, marital status, education level, experience, type, and residence.

## Study area

**Fig. 1:**
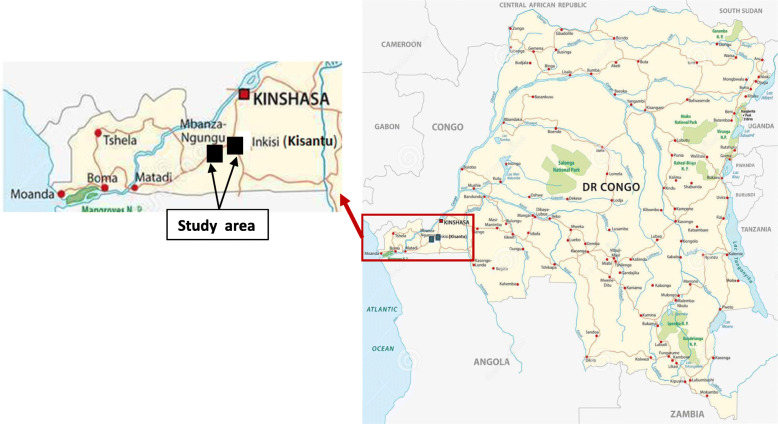
Study area location inside DR Congo (adapted from Lesniewski [25])

Our study was carried out in Kisantu and Mbanza-Ngungu towns as well as in the rural villages surrounding Mbanza-Ngungu, in Kongo-Central Province, Democratic Republic of Congo. Kisantu (also called Inkisi) is located at latitude 5° 08′ S and at longitude 15° 03′ E. Its altitude is estimated at 530 m above sea level (masl) [18]. Mbanza-Ngungu is located at latitude 5° 16′ S and at longitude 14° 5′ E. Its altitude ranges 500 to 750 masl [19]. The areas are characterized by a tropical Köppen AW_4_ climate with an average annual rainfall of 900 to 1500 mm and an annual average temperature of 25 °C [20] (Fig. 1).

Kisantu and Mbanza-Ngungu are predominantly inhabited by the Ntându and Ndibu ethnic groups, respectively, which are part of the Kongo people who inhabit an area that stretches from Congo-Brazzaville to Angola [21]. They share the same culture inherited from the ancient Kongo kingdom. The use of herbal medicine is well-rooted in their customs and habits. However, the Ntându culture has been strongly influenced by westernization and Christianity, whereas the Ndibu people have remained much attached to the traditional, ancestral Kongo religious practices [22, 23]. Farming and trading are the main economic activities of both ethno-linguistic groups [24]. Kisantu and Mbanza-Ngungu are interconnected, influence each other, and therefore share most socio-economic and cultural realities. They are both located in a Province where poverty is general and widespread. The health indicators for this province show a very worrying situation. These include the low coverage and precariousness of the health system (one general reference hospital for 126,700 inhabitants, one doctor for 17,356 inhabitants, one pharmacist for 131,069 inhabitants, one hospital bed for 514 inhabitants, one reference health center for 50,013 inhabitants) and low utilization of health services (49% for curative care) [20].

## Methods

### Data collection

We distinguished three types of herbal therapists according to their attitude: (1) traditional health practitioners (who heal using plant, animal, or mineral products); (2) herbalists (who know plants and use them for medicinal purposes), and (3) curing healers (who mainly use religious or other rites to heal). We defined them according to the Congolese [26] and Central African [27] law on herbal medicine. These herbal therapists are reputed to have in-depth knowledge on local plant properties and enjoy a certain notoriety among the public. However, all herbal therapists have their own area of expertise, do not necessarily use the same plants to treat diseases, and are distinguished in the art of curing diseases [28].

Ethnobotanical surveys were conducted between June 2017 to February 2018 and February to April 2019. A total of 188 informants, including herbalists, traditional health practitioners, and curing healers were interviewed. They were selected using the snowball method [10, 29, 30] and surveyed using semi-structured interviews. Following Thomas et al. [31], plant photos were used to complete interviews and to check the respondents’ ability to recognize the plants they use. We used photos depicted in Pauwels et al. [32] as well as their own photos taken during preparatory field visits in the study area.

Information on age, gender, marital status, education level, experience, socio-professional category, diseases treated, plants and plant organs used, medicinal pant growth location, preparation, and administration methods were collected during interviews. Following Sylva et al. [33], visits in the wild were carried out, accompanied by healers to the places where they harvest medicinal plants. Plant identification was done with the help of healers and by consulting studies from Gillet et al .[34], Nsimundele [35], Daeleman et al. [11], Budiongo [36], Mukoko [37], Pauwels [38], Malaisse [39], Kibungu [13], Nzuki [10], Latham et al. [40], and Nzenza et al. [41]. Voucher specimens of each species were collected and compared with species at the herbarium of Kisantu botanical garden or at the National Institute for Agronomic Studies and Research (INERA) at Kinshasa University (UNIKIN). Scientific names, in accordance with the APG IV system, were verified using websites such as Tela-Botanica (www.tela-botanica.org) or IPNI (International Plant Name Index: www.ipni.org).

### Ethnobotanical parameters measured

Relative importance attached to a given medicinal plant species in Kongo herbal medicine was calculated using UVs (medicinal use value) parameter by the formula of Phillips et al. [42] modified and used by Thomas et al. [43]:

1 $$ \mathrm{UVs}=\frac{\sum_{\mathrm{i}=1}^{\mathrm{n}}U\mathrm{is}}{\mathrm{n}\mathrm{s}} $$

in which UV_S_ is the use value of a given species s; *U*_is_ is the number of uses of species s mentioned by informant i; ns is the total number of informants.

As this parameter does not reflect the consensus of informants on medicinal plant use, we have also calculated for each species, the parameter IAR (informant agreement ratio) following Trotter and Logan [44], Thomas et al. [40] and Nzuki et al. [14]:

2 $$ \mathrm{IAR}=\frac{\mathrm{Nr}-\mathrm{Na}}{\mathrm{Nr}-1} $$

in which Nr is the total number of citations of the species and Na is the number of diseases treated by the species.

For each plant use, we calculated the ICF (informant consensus factor) parameter, which allows to verify informant agreement for a plant species in the treatment of a particular disease and to select species with an interesting therapeutic potential for phytochemical and pharmacological studies [44]. ICF was computed following Trotter and Logan [44]:

3 $$ \mathrm{ICF}=\frac{\mathrm{Nuc}-\mathrm{Nt}}{\mathrm{Nuc}-1} $$

in which Nuc is the number of citations of a particular disease; Nt is the number of species used for the treatment of that disease.

After documenting local uses, we selected species that seemed to be potentially effective for treating diseases and therefore merit phytochemical study. For this purpose, following to Heinrich [45], we considered species cited more than once for the treatment of a mentioned disease as potentially effective. To enable selection of such potentially interesting species, we defined and used the parameter species therapeutic potential (STP):

4 $$ STP\ \left(\%\right)=\frac{Ni-1}{Nti} $$

in which *N*_i_ is the number of informants who mentioned the use of a species for the treatment of a given disease, and *N*_ti_ is the total number of informants who mentioned any species for the treatment of that disease. The STP parameter allows the selection of the most frequently cited plants for the treatment of a given disease whereas the plants cited only once to be discarded. In other words, the STP allows to identify only the species with the highest level of consensus for each disease mentioned according to ICF to be selected.

### Data analysis

A synoptic table of inventoried medicinal plants and their use in Kisantu and Mbanza-Ngungu territories is presented in an additional file, available on https://data.mendeley.com/datasets/4cf2p3mgpc/1. MS Excel 2013 was used to process the data. Differences of traditional medicinal knowledge between Kongo social groups were analyzed using SPSS 25. Mann-Whitney and Kruskal-Wallis non-parametric tests as well as Poisson regression were employed to analyze differences in disease and medicinal plant knowledge between different social groups (distinguished according to gender, age, experience, education, marital status, categories, and residence). Results of Poisson regression, Mann-Whitney, and Kruskal-Wallis tests were considered as statistically significant for *p* values < 0.05. For ethno-cultural comparison between Kisantu (inhabited mainly by the Ntându ethnic group) and Mbanza-Ngungu (inhabited mainly by the Ndibu ethnic group) and between urban and rural respondents, we used the Rahman similarity index (RSI), which indicates the similarity of species used for treating the same diseases [46]. We assume that two communities are culturally closer if they use the same species to treat the same diseases.

5 $$ RSI\ \left(\%\right)=\frac{d}{a+b+c-d} $$

in which “a” is the number of unique species in community A, “b” is the number of unique species in community B, “c” is the number of common species in both A and B communities, and “d” is the number of common species in both A and B communities that are used to treat the same disease; *a* and *b* ≠ 0 and *c* and *d* ≥ 0.

## Results

### Informant profiles

The majority of informants were male (57.4%). Most of them were traditional health practitioners (81.9%). The sector is dominated by adults (72.3%) followed by the elderly (23.4%). Lowest number of respondents were situated in Mbanza-Ngungu urban area (20.7%) compared to Kisantu (43.1%) and Mbanza-Ngungu rural area (36.2%) respondents. Majority of respondents had at least received primary (33%) and secondary (45.5%) school education. Most respondents were married (70.2%) and had more than 10 years of experience (78.7%) with phytomedicine. The highest average number of cited species and diseases were recorded among elderly users (4.8 ± 4.2 species and 2.1 ± 1.9 diseases mentioned on average), practitioners with 5-10 years of experience (4.6 ± 4.4 species and 1.9 ± 1.9 diseases mentioned on average), male respondents (4.8 ± 3.9 species and 1.9 ± 1.9 diseases mentioned on average), curing healers (6.7 ± 5.6 species and 3.1 ± 2.6 diseases mentioned on average), married therapists (4.6 ± 3.9 species and 1.8 ± 1.9 diseases mentioned on average), respondents with at least secondary school education (4.7 ± 4.1 species and 2.1 ± 2.2 diseases mentioned on average), and therapists living in the Mbanza-Ngungu urban area (5.21 ± 4.4 and 2.5 ± 2.6) (Table 1).

**Table 1:** Informant sociological profiles and average (± SD) number of reported species and diseases

Factors	Category	No	%	No. of species	No. of disease
Gender	Woman	80	42.6	3.3±3.2	1.4±1.5
	Man	108	57.4	4.8±3.9	1.9±1.9
Age	Young	8	4.3	3±1.9	2.0±2.8
	Adult	136	72.3	4,0±3.6	1.6±1.7
	Old	44	23.4	4.8±4.2	2.1±1.9
School level	Illiterate	28	14.9	3.6±3.5	1.5±1.4
	Primary	62	33.0	4.2±3.5	1.5±1.4
	Secondary	78	41.5	4.7±4.1	2.1±2.2
	Superior	20	10.6	2.7±2.0	1.4±1.8
Experience (year)	0-5	12	6.4	2.5±1.6	1.0
	5-10	28	14.9	4.6±4.4	1.9±1.9
	> 10	148	78.7	4.2±3.6	1.8±1.9
Informants categories	Herbalist	13	6.9	1.9±1.5	1.0
	Curing healer	21	11.2	6.7±5.6	3.1±2.6
	traditional health practitioner	154	81.9	4.0±3.3	1.6±1.7
Marital stratus	Single	19	10.1	2.6±1.9	1.4±1.8
	Married	132	70.2	4.6±3.9	1.8±1.9
	Widower	37	19.7	3.5±2.8	1.6±1.5
Residence	Kisantu	81	43.1	4.7±4.2	1.9±1.9
	Mbanza-Ngungu urban area	39	20.7	5.2±4.4	2.5±2.6
	Mbanza-Ngungu rural area	68	36.2	2.9±1.8	1.0±0.1
	Total	188	100.0	4.2±3.7	1.7±1.8

### Taxonomic diversity

From a total of 231 plants inventoried, 227 species could be identified and classified in 192 genera and 79 families. Families representing most species were Fabaceae (27 species, i.e., 11.9%), Euphorbiaceae (13 species, i.e., 5.7%), Rubiaceae (12 species, i.e., 5.3%), Asteraceae and Lamiaceae (each with 11 species, i.e., 4.8%), and Solanaceae (10 species, i.e., 4.4%). The other 73 families were represented by less than 10 species (Table 2).

**Table 2:** Taxonomic diversity of medicinal plants in the study area

Family	Number of reported species	Share of reported species (%)	Number of genera	Share of genera (%)
Fabaceae	27	11.9	21	10.9
Euphorbiaceae	13	5.7	12	6.3
Rubiaceae	12	5.3	10	5.2
Asteraceae	11	4.8	11	5.7
Lamiaceae	11	4.8	6	3.1
Solanaceae	10	4.4	7	3.6
Poaceae	8	3.5	8	4.2
Apocynaceae	7	3.1	6	3.1
Malvaceae	7	3.1	7	3.6
Cucurbitaceae	5	2.2	5	2.6
Anacardiaceae	5	2.2	4	2.1
Moraceae	5	2.2	4	2.1
Zingiberaceae	5	2.2	4	2.1
Annonaceae	4	1.8	3	1.6
Araceae	4	1.8	4	2.1
Myrtaceae	4	1.8	3	1.6
Amaryllidaceae	3	1.3	1	0.5
Arecaceae	3	1.3	3	2.1
Cyperaceae	3	1.3	2	1.0
Dioscoreaceae	3	1.3	1	0.5
Phyllanthaceae	3	1.3	3	1.6
Other families (with < 3 species)	74	32.6	67	34.9
Total	227	100.0	192	100.0

### Medicinal plant use

**Fig. 2:**
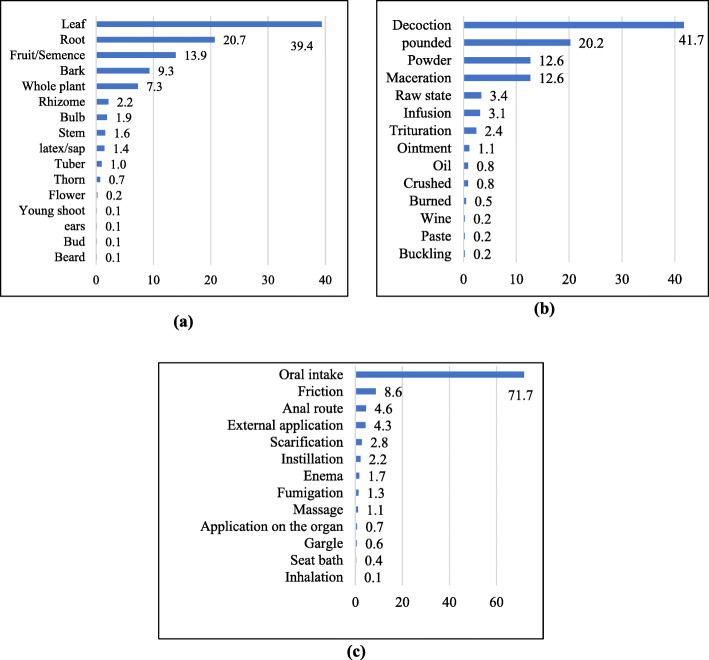
Share (%) in use reports of organs harvested (**a**), preparation (**b**), and administration methods (**c**) of plant medicines

A total of 337 plant medicine recipes have been identified, of which 203 are composed of at least two species, for the treatment of 103 diseases. Diseases most commonly treated by traditional medicine in the study area are hemorrhoids, hernias, and sexual weakness or impotence. The leaf (39.4%) was the most commonly used organ, whereas decoction (41.7%) and oral intake (71.7%) were the most common preparation and administration methods, respectively, in all use reports (Fig. 2).

**Fig. 3:**
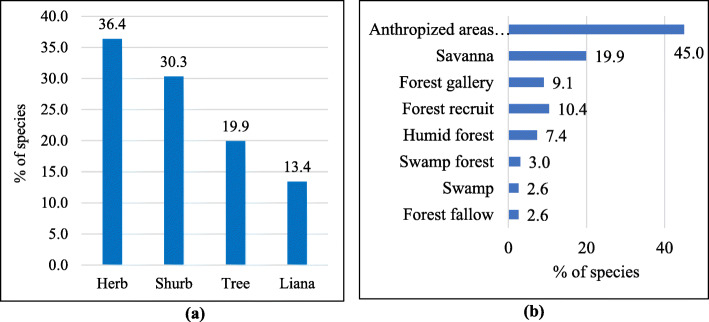
Share (%) in medicinal plant use reports of biological forms (**a**) and plant locations (**b**)

Herbs (36.4%) are the most widespread biological form, whereas anthropized areas such as fields, roadsides, homegardens, or in neighborhoods (45.0%) are the most frequent locations where inventoried medicinal plants are found (Fig. 3)

### Ethnobotanical data analysis

#### Relative importance of a given plant (UV_S_, IAR)

**Fig. 4:**
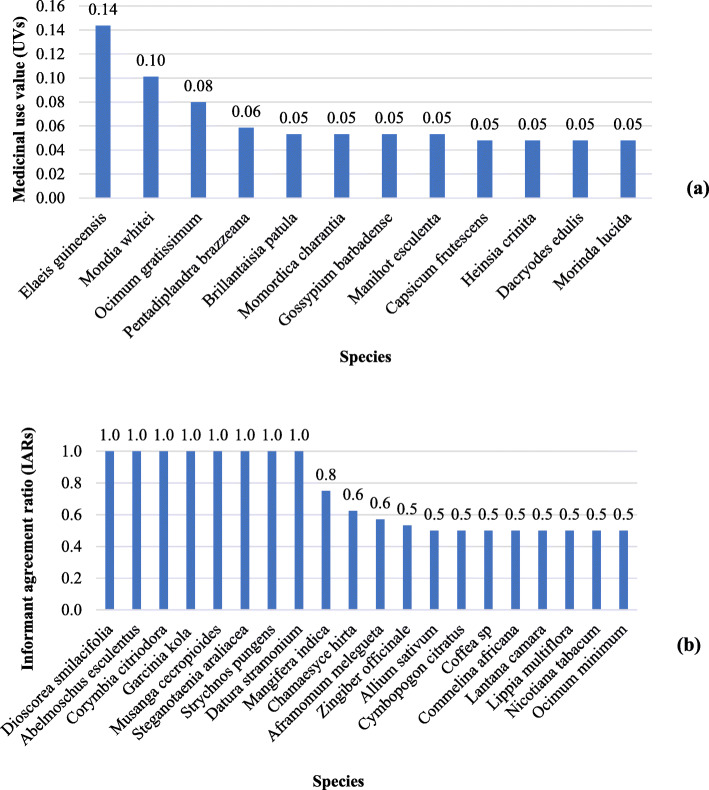
Ranking of most important medicinal plant species according to UV (**a**) and IAR (**b**)

Medicinal plant use value ranged from 0.01 to 0.14. *Elaeis guineensis* Jacq., (0.14), *Mondia whitei* (Hook. f.) Skeels (0.10), *Ocimum gratissimum* L., (0.08), and *Pentadiplandra brazzeana* Baillon (0.06) are the most important species in the traditional Kongo pharmacopeia, with UVs > 0.05 (Fig. 4a). The informant agreement on plant use ranged 0.1 to 1. *Dioscorea smilacifolia* De Wild. & T. Durand, *Abelmoschus esculentus* (L.) Moench, *Corymbia citriodora* (Hook.) K. D. Hill & L. A. S. Johnson, *Garcinia kola* Heckel, *Musanga cecropioides* R. Br., *Steganotaenia araliacea* Hochst, *Strychnos pungens* Soler., and *Datura stramonium* L. have the maximum IAR-value of 1 (Fig. 4b). They represent the species with the highest level of consensus for their use as a remedy for diabetes, cough, epilepsy, laryngitis, hernia, elephantiasis, hair yellowing, and tooth decay, respectively.

#### Informant consensus factor and species therapeutic potential

ICF ranged 0.05 to 0.44 and a total of 31 diseases have been highlighted. Diseases with ICF values ≥ 0.20 include hemorrhoids (0.44), amebiasis (0.43), itchy rash (0.42), poliomyelitis (0.36), intestinal parasitosis (0.33), sexual weakness or impotence (0.32), splenomegaly (0.29), laryngitis (0.27), rheumatism (0.25), otitis, (0.25), hernia (0.2), and cough (0.2) (

**Table 3:** Plant use consensus and species with high therapeutic potential

Diseases	ICF	Species with therapeutic potential
**Amoebiasis**	**0.43**	*Allium sativum* L.
		*Chamaesyce hirta* (L.) Millsp*.*
		*Elaeis guineensis* Jacq.
Anemia	0.1	*Ochna afzelii* R.Br. ex Oliv.
Hookworm	0.17	*Allium cepa* L.
		*Allium sativum* L.
		*Piper nigrum* L*.*
Asthma	0.09	*Imperata cylindrica* (L.) P.Beauv
Dental caries	0.14	*Datura stramonium* L.
		*Elaeis guineensis* Jacq.
Headache	0.18	*Brillantaisia ​​patula* T. Anderson
		*Ocimum gratissimum* L.
Diabetes	0.05	*Abelmoschus esculentus* (L.) Moench
Chest or intercostal pain	0.1	*Eleusine africana* Kenn.-O'Byrne
Premature ejaculation	0.14	*Zingiber officinale* Roscoe
Elephantiasis and yellowing of the hair	0,17	*Strychnos pungens* Soler.
Epilepsy	0.19	*Aframomum melegueta* (Roscoe) K.Schum.
		*Allium cepa* L.
		*Garcinia kola* Heckel
		*Lippia multiflora* Moldenke
		*Mucuna pruriens* (L.) DC.
**Itchy skin rash**	**0.42**	*Brillantaisia patula* T. Anderson
		*Clerodendrum formicarum* Gürke
		*Commelina africana* L.
		*Mondia whitei* (Hook. f.) Skeels
		*Nymphaea lotus* L.
**Sexual weakness or impotence**	**0.32**	*Mondia whitei* (Hook. f.) Skeels
		*Newbouldia laevis* (P.Beauv.) Semble.
		*Heinsia crinita* (Afzel.) G.Taylor
		*Gardenia ternifolia* Schumach. & Thonn. Subsp.
		*Hymenocardia acida* Tul.
		*Coffea sp.*
		*Dioscorea smilacifolia* De Wild. &T.Durand
Madness	0.07	*Brillantaisia patula* T. Anderson
Chronic scabies with itching and stench	0.1	*Gossypium barbadense* L.
Gastritis	0.14	*Allium cepa* L.
		*Brillantaisia ​​patula* T. Anderson
		*Citrus limon* (L.) Burm.f.
		*Jatropha curcas* L.
**Hemorrhoids**	**0.44**	*Aframomum melegueta* (Roscoe) K.Schum.
		*Capsicum frutescens* L.
		*Mangifera indica* L.
		*Monodora angolensis* T. Anderson
		*Piper nigrum* L.
		*Zingiber officinale* Roscoe
**Hernia**	**0.2**	*Desmodium mauritianum* (Willd.) DC.
		*Gardenia ternifolia* Schumach. & Thonn. Subsp.
		*Hallea stipulosa* (DC.) J.-F.Leroy
		*Pentadiplandra brazzeana* Baillon
		*Sarcocephalus latifolius* (Sm.) E. A. Bruce
		*Sarcocephalus pobeguinii* Pobeg.
		*Steganotaenia araliacea* Hochst.
		*Xylopia aethiopica* (Dunal) A. Rich.
**Laryngitis**	**0.27**	*Bridelia ferruginea* Benth.
		*Kalaharia uncinata* (Schinz) Moldenke
		*Musanga cecropioides* R. Br.
		*Pentadiplandra brazzeana* Baillon
Malaria	0.17	*Artemisia annua* L.
Mastitis	0.05	*Momordica charantia* L.
Microfilariate	0.1	*Momordica charantia* L.
Migraine	0.15	*Elaeis guineensis* Jacq.
		*Eleusine africana* Kenn.-O'Byrne
		*Ocimum minimum* L.
Sciatic neuralgia	0.15	*Elaeis guineensis* Jacq.
		*Ocimum gratissimum* L.
**Otitis**	**0.25**	*Ocimum gratissimum* L.
**Poliomyelitis**	**0.36**	*Aframomum melegueta* (Roscoe) K.Schum.
		*Cymbopogon citratus* (DC.) Stapf
		*Cyperus articulatus* L.
		*Securidaca longepedunculata* Fresen.
Prostate	0.13	*Zea mays* L.
**Splenomegaly**	**0.29**	*Elaeis guineensis* Jacq.
		*Eleusine africana* Kenn.-O'Byrne
**Rheumatism**	**0.25**	*Aframomum melegueta* (Roscoe) K.Schum.
		*Capsicum frutescen* L.
		*Elaeis guineensis* Jacq.
		*Musa x paradisiaca* L.
		*Ocimum gratissimum* L.
		*Securidaca longipedunculata* Fresen.
**Cough**	**0.2**	*Cymbopogon citratus* (DC.) Stapf
		*Corymbia citriodora* (Hook.) K. D. Hill & L. A. S. Johnson
		*Lantana camara* L.
**Intestinal parasitosis**	**0.33**	*Zingiber officinale* Roscoe

Table 3). For each reported disease, we identified the species with the highest STP. A total of 54 plant species has thus been identified and considered as having effective therapeutic potential (Table 3). Hernia is the pathology with the highest number of reported medicinal plant remedies. *Elaeis guineensis* Jacq. is said to be used to treat the highest number of diseases including amoebiasis, dental caries, migraine, sciatic neuralgia, splenomegaly, and rheumatism (Table 3).

#### Medicinal knowledge

Despite the large number of common species (105) and diseases (40) that are reported in both territories, a low ethnocultural similarity (RSI) was observed (16.7%) between respondents from Kisantu and Mbanza-Ngungu. Most similarity was observed in the use of common species for similar diseases between respondents from Kisantu and those of Mbanza-Ngungu rural areas (RSI = 15.6%), followed by similarity between respondents of Mbanza-Ngungu urban and rural areas, respectively (RSI = 8.1%). Ethnocultural similarity of medicinal plant knowledge was lowest (RSI = 5.7%) between respondents from Kisantu and those from Mbanza-Ngungu urban areas (RSI = 5.7%) (Table 4).

**Table 4:** Ethnomedicinal cultural similarity between Kisantu and Mbanza-Ngungu territories

Ethnic groups/regions	Parameter	Kisantu	Mbanza-Ngungu (Urban area)	Mbanza-Ngungu (Rural area)	Number of species inventoried	Number of diseases recorded
Kisantu	a	-	60	13		
	b	-	89	86		
	c	-	74	77	163	71
	d	-	12	24		
	e	-	32	27		
	RSI	-	5.7%	15.8		
Mbanza-Ngungu (urban & rural areas)	a	58	-	-		
	b	68	-	-		
	c	105	-	-	173	72
	d	33	-	-		
	e	40	-	-		
	RSI	16.7%	-	-		
Mbanza-Ngungu (urban area)	a	89	-	39		
	b	60	-	83		
	c	74	-	51	134	52
	d	12	-	13		
	e	32	-	23		
	RSI	5.7%	-	8.1%		
Mbanza-Ngungu (rural area)	a	86	83	-		
	b	13	39	-		
	c	77	51	-	90	43
	d	24	13	-		
	e	27	23	-		
	RSI	15.8%	8.1%	-		

a (number of unique species in community A), b (number of unique species in community B), c (number of common species in both A and B communities), d (number of common species used for the same diseases in both A and B communities), e (number of equal diseases reported in both A and B communities) and RSI (Rahman similarity index).

Traditional Kongo medicinal knowledge (based on number of reported medicinal species and diseases) was found to be independent of age, education, experience, and marital status (*p* > 0.05), but was significantly (*p* < 0.05) influenced by informant gender, quality, and residence.

Mean number of species cited was found to be significantly (*p* < 0.05) different between (1) curing healers, herbalists, and traditional health practitioners, with curing healers citing 1.46 times more species (*b* = 0.383; S.E = 0.1056, *p* = 0.00 than traditional health practitioners, and traditional practitioners citing 0.44 times more species than herbalists (*b* = −0.812; S.E = 0.216; *p* = 0.00); (2) informants from cities (in both Kisantu and Mbanza-Ngungu) and those from villages, with no significant differences (*p* > 0.05) between Kisantu and Mbanza-Ngungu cities and with informants from Kisantu and Mbanza-Ngungu significantly citing 53 times (*b* = 0.426; S.E = 0.977; *p* = 0.00) and 1.76 times (*b* = 0.565; S.E = 0.999; *p* = 0.00) more species, respectively, than informants from villages near Mbanza-Ngungu, and (3) men and women, with men citing 0.75 times more species than women (*b* = 0.290; S.E = 0.764; *p* = 0.00) (Table 5).

**Table 5:** Poisson regression model of the average number of species and diseases cited by the different social groups

Dependent	Factor	B (estimated coefficient)	Standard Error	*p*-value.	Exp(B) (exponentiated values)
Number of species	(Constant)	1.191	0.0769	.000	3.289
	Female	-0.29	0.0764	.000	0.749
	Male	0^a^	.	.	1
	Kisantu (urban)	0.426	0.0977	.000	1.53
	Mbanza-Ngungu urban	0.565	0,0999	.000	1.76
	Mbanza-Ngungu rural	0^a^	.	.	1
	Herbalists	-0.812	0.2165	.000	0.444
	Curing healer	0.383	0.1056	.000	1.467
	Traditional health practitioners	0^a^	.	.	1
	(Scale)	1^b^			
Number of diseases	(Constant)	0.132	0.1288	0.304	1.142
	Female	-0.279	0.1181	0.018	0.756
	Male	0^a^	.	.	1
	Kisantu (urban)	0,52	0.1616	0.001	1.682
	Mbanza-Ngungu urban	0.874	0.1576	0	2.397
	Mbanza-Ngungu rural	0^a^	.	.	1
	Herbalists	-0.467	0.3005	0.12	0.627
	Curing healer	0.574	0.1595	0	1.774
	Traditional health practitioners	0^a^	.	.	1
	(Scale)	1^b^			

^a^set to 0; ^b^Fixed display value

There were no significant differences between the mean number of diseases cited by curing herbalists and by traditional health practitioners. The number of diseases cited by curing healers was significantly (*p* < 0.05) different from those cited by herbalists and traditional health practitioners. The number of diseases cited by traditional health practitioners was 0.63 times higher than that of herbalists, whereas curing healers significantly cited 1.77 times more diseases than traditional health practitioners. The mean number of cited diseases was significantly different between informant residence (*p* < 0.05). Informants from Kisantu (*b* = 0.520; S.E = 0.1616; *p* = 0.01) and Mbanza-Ngungu (*b* = 0.874; S.E = 0.1576; *p* = 0.00) significantly cited respectively 1.68 and 2.40 times more diseases than informants from villages near Mbanza-Ngungu. To conclude, the mean number of cited diseases was also significantly different between men and women (*p* < 0.05), with men citing 0.76 times more diseases than women.

## Discussion

### Medicinal plant use

The Kongo people possess a rich and diversified ancestral medicinal knowledge, irrespective of their level of education, gender or marital status, or whether they live in cities or in the countryside. Of all 231 medicinal plant species inventoried in our study, 135 were also identified in ethnomedicinal studies from Nzuki et al. [14], 170 from Kibungu [13], and 70 from Nsimundele [35] in the same Kongo-Central Province. The predominance of taxa in the Fabaceae family corroborates observations of Amujoyegbe et al. [47] (Nigeria), Ribeiro et al. [48] (Brazil), and Ong et al. [49] (India and Bangladesh). The medicinal use of taxa in this family can probably be explained by the bioactive elements they contain, including tannins, alkaloids, coumarins, steroids, saponosides, flavonoids, and isoflavonoids [50].

The predominant medicinal plant part (leaves), botanical form (herbs), preparation (decoction), and administration (oral intake) method used were also observed in earlier medicinal plant studies [51–54]. The widespread medicinal use of leaves is probably due to the fact that they are easily and conveniently harvested [55] but also because they are the site par excellence of biosynthesis and storage of secondary metabolites, responsible for biological plant properties [56, 57].

The fact that vegetation has become highly disturbed by human activities in the region, can explain why most cited medicinal plants are herbaceous species. Abandoned fields, rudimentary environments, and trampled areas are quickly colonized by herbs to the detriment of forests and savannahs, which require a long transition period to regenerate [58].

The common practice of decoction as a medicinal plant preparation method can be explained by the fact that it is an easy way to collect the medicinally active principle compounds and to mitigate or eliminate toxic substances in certain medicinal plants [59]. The frequent use of oral absorption as medicinal plant administration route can be linked to the fact that it is fast and provides a large effective surface area for absorption of the drug’s active components [60]. Once absorbed, the drug passes through the intestinal wall and the liver before being transported to the target site by the bloodstream [61, 62].

### Local importance of plants and their consensus of use in disease treatment

Our findings indicate that 12 (5.2 %) or 20 (8.7%) of all inventoried medicinal plants are the most medically important species of the study area according to UV_S_ (> 0.05) and IAR (≥ 0.5), respectively.

The 12 diseases ranked as most important according to their ICF values have low ICF values when compared to those reported by other authors who performed ethnobotanical studies with the same Kongo people in other areas. This is the case, e.g., sexual weakness (ICF, 0.71) reported in Kinshasa [63] or for intestinal parasitosis (ICF, 0.48), rheumatism (ICF, 0.47), or otitis (ICF, 0.4) in Uíge Province, northern Angola [17]. Low ICF values could be explained by the tendency of phytotherapists to keep their knowledge secret from each other [64, 65].

The common medicinal use of *Elaeis guineensis* Jacq. is linked with the diverse products that are obtained from it, including oil, wine, and salt (palm inflorescence reduced to ashes) [66]. These extracts are used respectively as (1) an excipient for the preparation of vegetable ointments; (2) a maceration liquid to enhance the action of certain drugs with aphrodisiac and galactogenic properties; (3) an addition in many preparations to facilitate the absorption of the drug; or to reduce and preserve certain preparations in powder form. According to Raymond-Hamet [67], the ash salts of *Elaeis guineensis* Jacq. would release alkaloids from the plants used in the various preparations, which would explain why they are so frequently used by natives in multiple medications.

### Traditional medicinal knowledge among social and ethnic groups

Analyses on the influence of social factors on medicinal plant use indicated that male, married, adult, literate, experimented, and urban respondents were the most represented among phytotherapists. These findings are in line with those of Nzuki [10] (Mbanza-Ngungu) for gender, age, literacy, and respondent residence and with Ladoh-yemeda et al. [68] (Cameroun) for experience.

Descriptive statistics showed that curing healers, male therapists, adults and the elderly, married, educated (with secondary education), and urban respondents reported a higher average number of species and diseases than other respondent categories. Similar findings were obtained by Dapar et al. [69] for men, adults, and elderly, married and secondary-school-educated therapists as well as by Sanga [70] for therapists living in urban areas. Nzuki [10], however, found that in Mbanza-Ngungu, rural therapists may have more knowledge than urban therapists. This difference may be due to the fact that Nzuki’s observations are based on the comparison between urban and rural healers who use more than 10 medicinal plants rather than comparing the average number of plants used by both healer categories.

The high number of common species and diseases that are reported in both Kisantu and Mbanza-Ngungu (including their neighboring villages), may be linked to the fact that the two territories belong to the same Guineo Congolese phytogeographical region [71] and share more or less the same ecoclimatic and edaphic conditions and common realities [10]. Low similarity indices are often linked to a high diversity of ethnic groups in a certain area [72], globalization [73], the use of the same species for a wide variety of diseases, or to the limited cultural exchanges between the studied ethnolinguistic groups [74].

It was found that medicinal knowledge (average number of medicinal species and diseases reported) was significantly different between male and female respondents and between the different healer and residence categories, but not between the different healers’ marital status, age, or education categories.

Similar results were found by Dapar et al. [69] for gender and healer categories. However, the latter authors found that age, education, and marital status influenced respondent’s medicinal knowledge whereas there was no influence of their residence location.

Men are more knowledgeable about medicinal plants than women, probably because in traditional society, knowledge is generally passed on to men, more specifically to the family elder brother, providing them with a certain power in the family and notoriety in the society [75].

The high medicinal knowledge of urban phytotherapists can probably be linked to urban phytotherapists who make false claims about their competence, thereby taking advantage of naive patients who are often destitute and in desperate search of medical treatment. These phytotherapists, driven by profit generation, might then prescribe medicinal plant treatments, that do not always correspond to accurate ancestral knowledge. The high medicinal knowledge of curing healers can be associated to the use of medicinal plants for both physical and mental health problems [76].

## Conclusion

Kongo herbal medicine is rich in medicinal plant species. Some have high medicinal value, whereas others seem to have interesting therapeutic potential for certain diseases. It is essential to produce and conserve these important species. Conservation can be achieved ex situ through cultivation in fields, homegardens, or plantations, or in situ by maintaining and protecting their natural ecosystems. Potentially therapeutic herbal medicines should be subjected to phytochemical analysis in order to find more evidence for their true medicinal value. This should enable the Kongo people to take full social and economic advantage of their knowledge.

## Supplementary Information

**Additional file 1.** Synoptic table of medicinal plants and their use in Kisantu and Mbanza-Ngungu territories. Available on https://data.mendeley.com/datasets/4cf2p3mgpc/1; DOI: 10.17632/4cf2p3mgpc.1.

## Data Availability

All data can be obtained by request to the corresponding author. All voucher specimens are deposited at the Herbarium of the National Institute for Agronomic Studies and Research of Kinshasa University and will be deposited at the Botanical Garden of Meise in Belgium as soon as suitable conditions are established.

## Abbreviations

UV_S_: Use medicinal value
IAR: Informant agreement ratio
FIC: Informant consensus factor
STP: Species therapeutic potential
DRC: Democratic Republic of Congo
Iv: Vulnerability index
INERA: National Institute for Agronomic Studies and Research
UNIKIN: Kinshasa University
APG: Angiosperm Phylogeny Group
IPNI: International Plant Names Index
