# Correction to: Ethnobotanical characterization of medicinal plants used in Kisantu and Mbanza-Ngungu territories, Kongo-Central Province in DR Congo

**DOI:** 10.1186/s13002-021-00436-1

**Published:** 2021-02-05

**Authors:** Kibungu Kembelo Pathy, Nzuki Bakwaye Flavien, Belesi Katula Honoré, Wouter Vanhove, Patrick Van Damme

**Affiliations:** 1 Department of Environmental Sciences, Kinshasa University (UNIKIN), Kinshasa, XI BP 127, Democratic Republic of the Congo; 2 Laboratory of Tropical and Subtropical Agriculture and Ethnobotany, Ghent University, Coupure Links 653, B-9000 Ghent, Belgium; 3 Faculty of Tropical AgriSciences, Czech University of Life Sciences Prague, Kamycka 129, Prague 6 – Suchdol, 165 21 Prague, Czech Republic

**Correction to: J Ethnobiology Ethnomedicine 17, 5 (2021)**

**https://doi.org/10.1186/s13002-020-00428-7**

Following publication of the original article [1], it came to the attention of the journal that the fifth author’s name had been miswritten as ‘Van Damme Patrick’; the name should be written as ‘Patrick Van Damme’.

The name has been corrected in the published article and the corrected name may be found in this correction article, too.
