# Supplementary material for: Ethnobotanical characterization of medicinal plants used in Kisantu and Mbanza-Ngungu territories, Kongo-Central Province in DR Congo
Source: J Ethnobiol Ethnomed. 2021 Jan 23;17:5. doi: 10.1186/s13002-020-00428-7 (PMC7824950; doi:10.1186/s13002-020-00428-7)
Supplement: Supplementary file 1 — Additional file 1. Synoptic table of medicinal plants and their use in Kisantu and Mbanza-Ngungu territories. Available on https://data.mendeley.com/datasets/4cf2p3mgpc/1; DOI: 10.17632/4cf2p3mgpc.1. [file 13002_2020_428_MOESM1_ESM.pdf]

Synoptic table of medicinal plants and their use in Kisantu and Mbanza-Ngungu terroirs
(in which a “Kisantu”, B “Mbanza-Ngungu urban area and, c “Mbanza-Ngungu rural area”).

**Species**	**Local name**	**Botanic family**	**Recording area**	**UV_S**	**IAR**	**Disease**	**Organ**	**Method of preparation**	**Method of administration**	**Morpho-logy**	**Harvested area**
Abelmoschus esculentus (L.) Moench	Dongodongo	*Malvaceae*	a, b	0.01	1.00	Diabetes	Fruit/semence	Powder, maceration	Oral intake	Herb	Field, home garden
Abrus precatorius subsp. *africanus* Verdc.	Ngenguba	*Fabaceae*	a, c	0.01	0.00	Cough	Whole plant	Decoction	Oral intake	Liana	Savanna
						Madness	Root	Pounded			
Acanthospermum hispidum DC.	Madiata nzau	*Asteraceae*	a, b, c	0.02	0.00	Pallor or dry skin, intermittent sever fevers, continuous diarrhea and loss of weigth of the baby due to infidelity by one of the parents (Sanga)	Leaf	Powder	Scarification	Herb	Ruderal
						Tooth decay		Pounded	Application on the organ		
						Appendicitis		Decoction	Oral intake		
						Zoster		Powder	External application		
Adenia cissampeloides (Planch. Ex Benth.) Harms	Mupembe	*Passifloraceae*	b	0.01	0.00	Gastritis	Root	Decoction	Oral intake	Liana	Forest recruit
Aframomum alboviolaceum K.Schum.	Kitundubila, Ntundulu	*Zingiberaceae*	a, b, c	0.04	0.00	Microfilariate	Root	Pounded	External application	Herb	Savanna
						Hernia	Stem	Decoction	Oral intake		
						Paralysis	Root, Stem	Pounded	Scarification		
						Bronchitis	Leaf	Pounded	Oral intake		
						Rheumatism	Root	Decoction	Friction		
						Interruption of the menstruation without being pregnant	Root	Pounded	Oral intake		
						Back pain	Fruit/semence	Pounded	Anal route		
Aframomum melegueta (Roscoe) K. Schum.	Mondongo, Ndungu zi nzô	*Zingiberaceae*	a, b, c	0.04	0.57	Rheumatism	Fruit/semence, leaf	Powder	Scarification	Herb	Field, home garden
						Hemorrhoids	Fruit/semence	Pounded, maceration	Oral intake, anal route		
						Polio	Fruit/semence, leaf	Decoction	Oral intake		
						Hookworm	Fruit/semence	Powder	Oral intake		
						Epilepsy	Fruit/semence	Powder	Oral intake		
						Chest or intercostal pain	Fruit/semence	Pounded	Oral intake		
						Female infertility	Whole plant	Maceration	Oral intake		
Ageratum conyzoides L.	Mpata kasakula	*Asteraceae*	b	0.02	0.00	Tooth decay	Leaf	Pounded	Instillation	Herb	Ruderal
						Female infertility		Maceration	Seat bath		
						Back pain		Pounded	Friction		
						Bronchitis		Trituration	Oral intake		
Albizia adianthifolia var. *intermedia* (De Wild. & T.Durand) Villiers	Mululu	*Fabaceae*	a	0.01	0.00	Hookworm	Leaf	Decoction	Oral intake	Tree	Forest recruit
Alchornea cordifolia (Schumach.) Müll.Arg.	Kibunsi	*Euphorbiaceae*	a, b	0.03	0.20	Tooth decay	Leaf	Decoction	Gargle	Shrub	Humid forest
						Back pain		Pounded	Anal route		
						Hemorrhoids		Decoction, maceration	Igestion, anal route		
						Repeated abortion or frequent delivery of stillbirths		Decoction	Seat bath		
						Epilepsy		Raw state	Oral intake		

Synoptic table of medicinal plants and their use in Kisantu and Mbanza-Ngungu terroirs
(in which a “Kisantu”, B “Mbanza-Ngungu urban area and, c “Mbanza-Ngungu rural area”).

Species	Local name	Botanic family	Recording area	UV_S	IAR	Disease	Organ	Method of preparation	Method of administration	Morpho-logy	Harvested area
Allium cepa L.	Bola	*Amaryllidaceae*	a, b, c	0.03	0.43	Chest or intercostal pain	Bulb	Pounded	Friction	Herb	Field, home garden
						Hookworm		Infusion	Oral intake		
						Gastritis		Maceration	Oral intake		
						Madness		Decoction	Oral intake		
						Gastritis		Decoction	Oral intake		
						Hookworm		Infusion	Oral intake		
						Epilepsy	Leaf, Fruit/semence	Powder	Oral intake		
Allium fistulosum L.	Ndembi	*Amaryllidaceae*	a, c	0.01	0.00	Mastitis	Root	Pounded	Friction	Herb	Field, home garden
						Prostate	Whole plant	Maceration	Oral intake		
Allium sativum L.	Ayi	*Amaryllidaceae*	a, b, c	0.03	0.50	Amoebiasis	Bulb, Whole plant, Fruit/semence	Raw state, pounded, decoction	Oral intake, anal route	Herb	Field, home garden
						Premature ejaculation	Bulb	Pounded	Oral intake		
						Hookworm	Bulb	Decoction	Oral intake		
						Gastritis	Bulb	Decoction, maceration	Oral intake		
						Hemorrhoids	Bulb	Maceration	Oral intake		
						Hookworm	Bulb	Powder	Oral intake		
						Chickenpox	Bulb	Pounded	Oral intake		
						Hookworm	Bulb	Infusion	Oral intake		
Aloe congolensis De Wild. & T.Durand	Bà di nseki	*Aloaceae*	a, b, c	0.03	0.00	Hernia	Leaf	Decoction	Oral intake	Herb	Savanna
						Diabetes					
						Joint pain					
						Splenomegaly					
						Mastitis					
Alstonia congensis Engl.	Nsanga	*Apocynaceae*	b	0.01	0.00	Zoster	Stem	Decoction	Oral intake	Tree	Swamp forest
Amaranthus blitum L.	Nkuka bangulu	*Amaranthaceae*	b	0.01	0.00	Alcoholism and smoking	Leaf	Powder	Oral intake	Herb	Ruderal
Amphiblemma ciliatum Cogn.	Nsa masa	*Melastomata-ceae*	a, b	0.01	0.00	Rheumatism	Leaf	Decoction	Oral intake	Shrub	Ruderal
						Epilepsy		Maceration	Instillation		
Ananas comosus (L.) Merr.	Nanasi	*Bromeliaceae*	a, b, c	0.03	0.00	Sexual weakness or impotence	Fruit/semence	Pounded	Oral intake	Herb	Field, home garden
						Hernia	Fruit/semence	Decoction	Oral intake		
						Sciatic neuralgia	Fruit/semence	Infusion	Oral intake		
						Gastritis	Fruit/semence	Decoction, maceration	Oral intake		
						Yellow fever	Leaf	Pounded	Anal route		
Anchomanes difformis Engl.	Kikwa ki ba nkita	*Araceae*	b	0.01	0.00	Hepatic sirosis	Tuber	Decoction	Oral intake	Herb	Humid forest
						Hernia					
Anisophyllea quangensis Engl. ex Henriq.	Mfungu-Mfungu, Mbila esobe	*Anisophilla-ceae*	a	0.01	0.00	Asthma	Leaf	Powder	Oral intake	Shrub	Savanna
Annona muricata L.	Mbundu ngombi	*Annonaceae*	a, b	0.02	0.00	Pertussis	Leaf	Decoction	Oral intake	Shrub	Field, home garden
						Itchy skin rash		Pounded	External application		
						Cough		Decoction	Oral intake		
Annona senegalensis Pers. Subsp. Oulotricha Le Thomas	Lomboloka	*Annonaceae*	a, b	0.04	0.00	Tripanosomiasis	Root	Decoction	Oral intake	Shrub	Savanna
						Hemorrhoids	Root		Oral intake		
						Splenomegaly	Root		Anal route		
						Testicular disappearance	Root		Oral intake		
						Hernia	Root		Oral intake		
						Diabetes	Root		Oral intake		
						Anemia	Leaf		Oral intake		

Synoptic table of medicinal plants and their use in Kisantu and Mbanza-Ngungu terroirs
(in which a “Kisantu”, B “Mbanza-Ngungu urban area and, c “Mbanza-Ngungu rural area”).

Species	Local name	Botanic family	Recording area	UV_S	IAR	Disease	Organ	Method of preparation	Method of administration	Morpho-logy	Harvested area
Anthocleista schweinfurthii Gilg	Mpuku mpuku	*Gentianaceae*	b	0.01	0.00	Diarrhea	Root	Decoction	Anal route	Tree	Forest fallow
						Female infertility		Maceration			
Undefined	Anti-poison	*Undefined*	b	0.01	0.00	Poisoning	Bark	Raw state	Oral intake	Liana	Forest recruit
Antidesma rufescens Tul.	Fuitidi	*Euphorbiaceae*	b	0.01	0.00	Infections due to STI (sexually transmitted infections)	Bark	Decoction	Oral intake	Shrub	Savanna
Arachis hypogaea L.	Nguba	*Fabaceae*	a, c	0.01	0.00	Madness	Fruit/semence	Decoction	Oral intake	Herb	Field, home garden
						Diarrhea		Raw state			
Artemisia annua L.	Artemisia	*Asteraceae*	a	0.01	0.00	Malaria	Leaf	Decoction	Oral intake	Herb	Field, home garden
								Powder			
Bambusa vulgaris Schrad. ex JCWendl.	Tùutu	*Poaceae*	b	0.01	0.00	Tension	Young shoot	Decoction	Oral intake	Shrub	Forest recruit
Bidens pilosa L.	Nsolokoto	*Asteraceae*	a, c	0.02	0.00	Ache	Leaf	Trituration	Friction	Herb	Ruderal
						Sciatic neuralgia	Leaf	Decoction	Oral intake		
						Hemorrhoids	Fruit/semence	Crushed	Oral intake		
Boerhavia diffusa L.	Dibatabata, linioko ya tembe	*Nyctaginaceae*	a, b	0.02	0.00	Splenomegaly	Whole plant	Pounded	Anal route	Herb	Ruderal
						Furuncle	Leaf	Buckling	External application		
						Bronchitis	Leaf	Pounded	Oral intake		
Brassica oleracea L.	Nkoofi	*Brassicaceae*	a	0.01	0.00	Splenomegaly	Leaf	Pounded	Oral intake	Herb	Field, home garden
						Prostate		Decoction			
Bridelia ferruginea Benth.	Kimwindu	*Phyllanthaceae*	a, b, c	0.04	0.14	Gastritis	Root	Decoction	Oral intake	Shrub	Savanna
						Tuberculosis	Root	Decoction			
						Diabetes	Root	Decoction			
						Laryngitis	Root	Decoction			
						Rheumatism	Root	Decoction			
						Laryngitis	Root	Decoction			
						Interruption of the menstruation without being pregnant	Root	Pounded			
						Hemorrhoids	Bark	Decoction			
Brillantaisia patula T. Anderson	Lemba-lemba	*Acanthaceae*	a, b, c	0.05	0.31	Buruli ulcer	Leaf	Pounded	External application	Shrub	Field, home garden
						Heaviness in the head, seems to split in the baby	Leaf	Maceration	Oral intake		
						Headache	Leaf	Decoction	Oral intake		
						Madness	Root	Pounded	Oral intake		
						Joint pain	Leaf	Decoction	Oral intake		
						Chronic scabies with itching and stink	Leaf	Powder	External application		
						Acute nervousness	Whole plant	Pounded	Oral intake		
						Itchy skin rash	Leaf	Maceration	Oral intake		
						Headache	Leaf	Powder	Friction		
						Itchy skin rash	Leaf	Pounded	Oral intake		
						Gastritis	Leaf	Decoction, maceration	Oral intake		
						Alcoholism and smoking	Leaf	Powder	Oral intake		
						Gastritis	Leaf	Decoction	Oral intake		
						Madness	Leaf	Pounded	Oral intake		

Synoptic table of medicinal plants and their use in Kisantu and Mbanza-Ngungu terroirs
(in which a "Kisantu", B "Mbanza-Ngungu urban area and, c "Mbanza-Ngungu rural area").

**Species**	**Local name**	**Botanic family**	**Recording area**	**UV_S**	**IAR**	**Disease**	**Organ**	**Method of preparation**	**Method of administration**	**Morpho-logy**	**Harvested area**
Bryophyllum pinnatum Kurz.	Liyuki-yuki	*Crassulaceae*	a, c	0.01	0.00	Cough	Leaf	Pounded	Oral intake	Shrub	Ruderal
						Otitis		Trituration	Instillation		
Cajanus cajan (L.) Millsp.	Wàandu	*Fabaceae*	a, b	0.03	0.00	Migraine	Leaf	Pounded	Instillation	Shrub	Field, home garden
						Chronic scabies with itching and stink	Fruit/semence	Powder	Friction		
						Gonorrhea	Fruit/semence	Decoction	Oral intake		
						Skin rash	Fruit/semence	Maceration	Oral intake		
						Polio	Leaf	Decoction	Oral intake		
Caloncoba welwitschii Gilg	Kisani	*Salicaceae*	a	0.01	0.00	Chest or intercostal pain	Leaf	Pounded	Friction	Shrub	Forest recruit
Canarium schweinfurthii Engl.	Kibidi	*Burseraceae*	b	0.01	0.00	Migraine	Bark	Maceration	Enema	Tree	Forest gallery
Canna indica L.	Makombu kombu	*Cannaceae*	a	0.01	0.00	Lack of uvula causing the baby to cry continuously	Leaf	Maceration	Oral intake	Herb	Ruderal
Capsicum frutescens L.	Ndungu zi ntendi	*Solananceae*	a, b, c	0.05	0.27	Rheumatism	Leaf	Powder, ointment	Massage, scarification	Herb	Field, home garden
						Sexual weakness or impotence	Fruit/semence	Powder	Oral intake	Herb	Field, home garden
						Hemorrhoids	Fruit/semence, leaf	Pounded	Oral intake, anal route		
						Hernia	Root	Decoction	Oral intake		
						Chronic scabies with itching and stink	Leaf	Powder	Friction		
						Migraine	Leaf	Trituration	External application		
						Whitlow	Fruit/semence	Pounded	External application		
						Mastitis	Leaf	Pounded	Friction		
						Female infertility	Fruit/semence	Maceration	Oral intake		
Carica papaya L.	Dipapayi	*Caricaceae*	a, b, c	0.03	0.00	Amoebiasis	Fruit/semence	Pounded	Anal route	Tree	Field, home garden
						Tooth decay	Fruit/semence	Raw state	Fumigation		
						Sciatic neuralgia	Root	Decoction	Friction		
						Epilepsy	Leaf	Powder	Oral intake		
						Mastitis	Root	Pounded	Friction		
						Malaria	Leaf	Infusion	Oral intake		
Catharanthus roseus (L.) G.Don	vinca	*Apocynaceae*	a	0.01	0.00	Amoebiasis	Leaf, root	Decoction	Oral intake	Herb	Ruderal
Ceiba pentandra (L.) Gaertn.	Mfuma	*Malvaceae*	b	0.01	0.00	Pneumonia	Bark	Pounded	Friction	Tree	Forest gallery
						Epilepsy		Maceration	Oral intake		
Cenchrus purpureus (Schumach.) Morrone	Madiadia	*Poaceae*	b	0.01	0.00	Madness	Leaf	Pounded	Oral intake	Herb	Swamp
Chamaesyce hirta (L.) Millsp.	Kimvumina nkombo, Kula ntesi	*Euphorbiaceae*	a, c	0.02	0.63	Amoebiasis	Leaf, whole plant	Decoction, raw state, pounded	Oral intake	Herb	Ruderal
						Breastfeeding	Whole plant	Decoction	Oral intake		
						Hemorrhoids	Whole plant	Maceration	Oral intake, anal route		
						Hookworm	Whole plant	Infusion	Oral intake		
Chenopodium ambrosioides L.	Nkasa kindongo	*Chenoponideae*	a, c	0.02	0.00	Tooth decay	Whole plant	Decoction	Oral intake	Herb	Ruderal
						Cough	Whole plant	Pounded	Oral intake		
						Amoebiasis	Leaf, stem	Decoction	Oral intake		
						Rheumatism	Whole plant	Decoction	Friction		
Chromolaena odorata (L.) RMKing & H.Rob.	Kolera	*Asteraceae*	a	0.01	0.00	Cough	Leaf	Decoction	Oral intake	Herb	Savanna

Synoptic table of medicinal plants and their use in Kisantu and Mbanza-Ngungu terroirs
(in which a "Kisantu", B "Mbanza-Ngungu urban area and, c "Mbanza-Ngungu rural area").

**Species**	**Local name**	**Botanic family**	**Recording area**	**UV$_S$**	**IAR**	**Disease**	**Organ**	**Method of preparation**	**Method of administration**	**Morpho-logy**	**Harvested area**
Cinnamomum verum J.Presl	Arbre du bonheur	*Lauraceae*	a	0.01	0.00	Premature ejaculation	Bark	Decoction	Oral intake	Tree	Humid forest
Cissus aralioides (Welw. Ex Baker) Planch.	Mbua mpimbidi, kindamina	*Vitaceae*	b, c	0.01	0.00	Elephantiasis and yellowing of the hair	Leaf	Decoction	Oral intake	Liana	Humid forest
						Malnutrition and kwashiorkor					
Cissus rubiginosa (Welw. Ex Baker) Planch.	Mumpongo mpongo	*Vitaceae*	a	0.01	0.00	Back pain	Leaf	Decoction	Oral intake	Liana	Forest fallow
						Amoebiasis		Infusion			
Citrullus lanatus (Thunb.) Matsum. Et Nakai	Mbika ntetu	*Cucurbitaceae*	b	0.01	0.00	Epilepsy	Leaf	Raw state	Oral intake	Liana	Field, home garden
Citrus limon (L.) Osbeck	Lala di ngani	*Rutaceae*	a, b, c	0.03	0.17	Pertussis	Leaf	Decoction	Oral intake	Shrub	Field, home garden
						Buruli ulcer	Fruit/semence	Raw state	Enema		
						Sciatic neuralgia	Fruit/semence	Decoction	Friction		
						Anemia	Fruit/semence	Decoction	Oral intake		
						Gastritis	Fruit/semence	Decoction, infusion	Oral intake		
						Mastitis	Fruit/semence	Pounded	Friction		
Citrus reticulata Blanco	Mandeleni	*Rutaceae*	b	0.01	0.00	Female infertility	Root	Maceration	Seat bath	Shrub	Field, home garden
Clematis hirsuta Guill. & Perr.	Nkonka-ntu	*Ranunculaceae*	a	0.02	0.00	Headache	Leaf	Decoction	Oral intake	Liana	Forest recruit
						Migraine	Leaf	Pounded	Friction		
						Splenomegaly	Root	Maceration	Oral intake		
						Prostate	Leaf	Decoction	Oral intake		
Clerodendrum formicarum Gürke	Makuku ma tatu	*Lamiaceae*	a, b, c	0.03	0.20	Asthma	Leaf	Powder	Oral intake	Herb	Ruderal
						Pallor or dry skin, intermittent sever fevers, continuous diarrhea and loss of weigth of the baby due to infidelity by one of the parents (Sanga)	Leaf	Powder	Scarification		
						Headache	Leaf	Crushed	Friction		
						Itchy skin rash	Leaf	Maceration, pounded	Oral intake		
						Epilepsy	Leaf	Raw state	Oral intake		
Clerodendrum splendens G.Don	Kindangolo	*Lamiaceae*	a	0.01	0.00	Polio	Leaf	Powder	Scarification	Liana	Forest recruit
Cocos nucifera L.	Nkandi mputu, Kokoti	*Arecaceae*	a	0.01	0.00	Sexual weakness or impotence	Fruit/semence	Pounded	Oral intake	Tree	Field, home garden
Coffea sp.	Kafi	*Rubiaceae*	a, b	0.01	0.50	Sexual weakness or impotence	Fruit/semence	Powder	Oral intake	Shrub	Field, home garden
						Hernia					
Cogniauxia podolaena Baill.	Kisakamba	*Cucurbitaceae*	a	0.01	0.00	Frigidity and narrowing of the vagina	Root	Decoction	Oral intake	Liana	Ruderal
Cola acuminata (P.Beauv.) Schott & Endl.	Kasu	*Malvaceae*	a, b, c	0.04	0.14	Heaviness in the head, seems to split in the baby	Fruit/semence	Maceration	Oral intake	Shrub	Field, home garden
						Hemorrhoids		Maceration			
						Premature ejaculation		Infusion			
						Epilepsy		Powder			

Synoptic table of medicinal plants and their use in Kisantu and Mbanza-Ngungu terroirs
(in which a "Kisantu", B "Mbanza-Ngungu urban area and, c "Mbanza-Ngungu rural area").

Species	Local name	Botanic family	Recording area	UV_S	IAR	Disease	Organ	Method of preparation	Method of administration	Morpho-logy	Harvested area
						Skin rash		Maceration			
						Sexual weakness or impotence		Infusion			
						Chest or intercostal pain		Pounded			
Combretum racemosum P. Beauv.	Nsumbila	*Combretaceae*	b	0.01	0.00	Healing	Leaf	Trituration	External application	Liana	Forest gallery
						Pneumonia		Pounded	Friction		
Commelina africana L.	Nlakisi	*Commelinaceae*	a, b, c	0.01	0.50	Itchy skin rash	Leaf	Maceration, pounded	Oral intake	Herb	Ruderal
						Migraine		Maceration	Enema		
Corymbia citriodora (Hook.) K. D. Hill & L. A. S. Johnson	Bikalipitusu	*Myrtaceae*	b	0.01	1.00	Cough	Leaf	Decoction	Oral intake	Tree	Forest gallery
Costus lucanusianus J.Braun et K.Schum.	Maboso boso	*Costaceae*	a, b, c	0.02	0.00	Asthma	Whole plant	Pounded	Oral intake	Herb	Humid forest
						Hiccup	Leaf	Pounded			
						Chickenpox	Whole plant	Pounded			
						Madness	Root	Decoction			
Costus phyllocephalus K. Schum.	Minkeni	*Costaceae*	a, b, c	0.04	0.00	Asthma	Whole plant	Pounded	Oral intake	Herb	Humid forest
						Enuresis	Root	Decoction	Oral intake		
						Tension	Whole plant	Pounded	Oral intake		
						Female infertility	Stem	Decoction	Oral intake		
						Breastfeeding	Stem	Decoction	Oral intake		
						Acute nervousness	Whole plant	Pounded	Oral intake		
						Itchy skin rash	Leaf	Pounded	External application		
						Mastitis	Whole plant	Pounded	Oral intake		
Craterispermum schweinfurthii Hiern	Muntoma-ntoma	*Rubiaceae*	a	0.01	0.00	Mastitis	Root	Decoction	Oral intake	Shrub	Forest recruit
Crossopteryx febrifuga (Afzel. Ex G. Don) Benth.	Mvala, Kigala	*Rubiaceae*	a, b, c	0.04	0.00	Elephantiasis and yellowing of the hair	Leaf et Root	Decoction	Oral intake	Shrub	Savanna
						Ovarian or tubal inflammation	Root	Decoction	Oral intake		
						Hernia	Bark	Infusion	Anal route		
						Laryngitis	Bark	Decoction	Oral intake		
						Rheumatism	Bark	Decoction	Oral intake		
						Interruption of the menstruation without being pregnant	Bark	Pounded	Oral intake		
						Female infertility	Bark	Maceration	Oral intake		
						Splenomegaly	Leaf	Raw state	Oral intake		
Croton mubango Müll.Arg.	Mbangu mbangu	*Euphorbiaceae*	b, c	0.02	0.00	Testicular disappearance	Bark	Pounded	Oral intake	Tree	Field, home garden
						Gastritis		Decoction			
						Dermatosis		Powder			
Cucurbita maxima Duchesne	Mbika malenge	*Cucurbitaceae*	c	0.01	0.00	Tuberculosis	Leaf	Maceration	Oral intake	Liana	Field, home garden
						Microfilariate		Decoction			
Culcasia scandens P. Beauv.	Lulama lama	*Araceae*	b	0.01	0.00	Alcoholism and smoking	Leaf	Powder	Oral intake	Herb	Humid forest
Curcuma longa L.	Kingoni	*Zingiberaceae*	b	0.01	0.00	Madness	Rhizome	Decoction	Oral intake	Herb	Field, home garden
						Hernia					

Synoptic table of medicinal plants and their use in Kisantu and Mbanza-Ngungu terroirs
(in which a “Kisantu”, B “Mbanza-Ngungu urban area and, c “Mbanza-Ngungu rural area”).

Species	Local name	Botanic family	Recording area	UV_S	IAR	Disease	Organ	Method of preparation	Method of administration	Morpho-logy	Harvested area
Cymbopogon citratus (DC.) Stapf	Sinda di mputu	*Poaceae*	a, b, c	0.02	0.50	Cough	Leaf	Pounded, decoction	Oral intake	Herb	Field, home garden
						Polio		Decoction			
						Hookworm		Powder			
Cyperus articulatus L.	Lusaku saku	*Cyperaceae*	a, b, c	0.03	0.33	Hemorrhoids	Root	Infusion, macération	Oral intake	Herb	Swamp
						Enuresis	Root	Decoction			
						Polio	Root	Decoction			
						Skin rash	Root	Maceration			
						Hookworm	Root	Powder			
Cyperus papyrus L.	Bù, Nkuala	*Cyperaceae*	a	0.01	0.00	Migraine	Stem	Powder	Friction	Herb	Swamp
Dacryodes edulis (G.Don) H.J.Lam	Nsafu	*Burseraceae*	a, b, c	0.05	0.00	Rheumatism	Leaf	Powder	Scarification	Tree	Field, home garden
						Repeated abortion or frequent delivery of stillbirths	Leaf	Maceration	Enema		
						Tooth decay	Leaf	Decoction	Fumigation		
						Chronic scabies with itching and stink	Leaf	Powder	External application		
						Headache	Leaf	Powder	Friction		
						Nephritis	Leaf	Pounded	Oral intake		
						Testicular disappearance	Leaf	Decoction	Oral intake		
						Zoster	Leaf	Powder	External application		
						Hernia	Root	Decoction	Oral intake		
Datura stramonium L	36 oiseaux	*Solananceae*	a	0.01	1.00	Tooth decay	Fruit/semence	Burnt	Fumigation	Shrub	Field, home garden
Daucus carota L.	Kaloti	*Apiaceae*	a	0.01	0.00	Sexual weakness or impotence	Tuber	Pounded	Application on the organ	Herb	Field, home garden
Desmodium mauritianum (Willd.) DC.	Lunzila nzila	*Fabaceae*	a	0.02	0.25	Splenomegaly	Whole plant	Decoction	Anal route	Herb	Ruderal
						Hernia	Leaf, root		Oral intake		
						Pallor or dry skin, intermittent sever fevers, continuous diarrhea and loss of weigth of the baby due to infidelity by one of the parents (Sanga)	Leaf		Scarification		
						Hookworm	Leaf		Oral intake		
Desmodium velutinum subsp. Velutinum (Willd.) DC.	Lundondu	*Fabaceae*	a	0.01	0.00	Hernia	Root	Decoction	Oral intake	Herb	Ruderal
						Headache	Leaf	Crushed	Friction		
Dichrostachys cinerea (L.) Wight & Arn.	Nsendi vanga	*Fabaceae*	b	0.02	0.00	Poisoning	Leaf	Decoction	Oral intake	Shrub	Savanna
						Hookworm	Leaf	Powder			
						Gastritis	Root	Decoction			
Dioscorea cayenensis Lam.	Kisadi	*Dioscoreaceae*	a, b	0.02	0.00	Hernia	Thorn	Decoction	Oral intake	Liana	Field, home garden
						Sciatic neuralgia		Infusion			
						Hookworm		Powder			
Dioscorea smilacifolia De Wild. &T.Durand	Ngolo	*Dioscoreaceae*	a	0.01	1.00	Sexual weakness or impotence	Thorn<br>Fruit/semence	Powder	Oral intake	Liana	Humid forest
Dioscorea bulbifera L.	Nsoko ngamba, kimasoko	*Dioscoreaceae*	b	0.01	0.00	Diabetes	Tuber	Decoction	Oral intake	Liana	Field, home garden

Synoptic table of medicinal plants and their use in Kisantu and Mbanza-Ngungu terroirs
(in which a “Kisantu”, B “Mbanza-Ngungu urban area and, c “Mbanza-Ngungu rural area”).

**Species**	**Local name**	**Botanic family**	**Recording area**	**UV$_S$**	**IAR**	**Disease**	**Organ**	**Method of preparation**	**Method of administration**	**Morpho-logy**	**Harvested area**
Dissotis brazzae Cogn.	Ntongu ntongu	*Melastomataceae*	a	0.01	0.00	Rheumatism	Leaf	Decoction	Oral intake	Herb	Savanna
Dorstenia psilurus Welw.	Kintamba	*Moraceae*	b, c	0.02	0.00	Hookworm	Root	Decoction	Oral intake	Herb	Forest gallery
						Erectil malfunction		Decoction	Oral intake		
						Mastitis		Pounded	Friction		
Dracaena mannii Baker	Boma libala	*Asparagaceae*	b	0.01	0.00	Smallpox	Bark	Decoction	Anal route	Shrub	Forest gallery
Elaeis guineensis Jacq.	Bà di ngasi	*Arecaceae*	a, b	0.14	0.28	Pneumonia	Fruit/semence	Pounded	Friction	Tree	Field, home garden
						Splenomegaly	Fruit/semence	Oil, raw state, paste	Oral intake, scarification, anal route		
						Buruli ulcer	Fruit/semence	Pounded	External application		
						Amoebiasis	Fruit/semence	Raw state	Oral intake		
						Vitiligo	Fruit/semence	Oil	Oral intake, External application		
						Breast care to keep it upright	Fruit/semence	Powder	Scarification		
						Sexual weakness or impotence	Root	Pounded	Oral intake		
						Migraine	Fruit/semence	Pounded	Friction		
						Sciatic neuralgia	Fruit/semence	Pounded	Friction		
						Tooth decay	Fruit/semence	Burnt	Fumigation		
						Headache	Fruit/semence	Pounded	Friction		
						Ache	Fruit/semence	Pounded	Friction		
						Chest or intercostal pain	Fruit/semence	Pounded	Friction		
						Amoebiasis	Fruit/semence, sève/latex	Decoction, pounded	Oral intake		
						Sciatic neuralgia	Fruit/semence	Oil	Oral intake		
						Hemorrhoids	Fruit/semence	Ointment	Anal route		
						Premature ejaculation	Fruit/semence	Raw state	Oral intake		
						Testicular disappearance	Latex/sap	Decoction	Oral intake		
						Hernia	Latex/sap	Decoction	Oral intake		
						Madness	Latex/sap	Decoction	Oral intake		
						Chronic scabies with itching and stink	Latex/sap	Raw state	Friction		
						Diabetes	Latex/sap	Decoction	Oral intake		
						Frigidity and narrowing of the vagina	Latex/sap	Decoction	Oral intake		
						Whitlow	Fruit/semence	Pounded	External application		
						Rheumatism	Fruit/semence	Powder	Massage		
						Asthma	Leaf	Decoction	Oral intake		
						Epilepsy	Latex/sap	Maceration	Oral intake		
						Yellow fever	Latex/sap	Pounded	Anal route		
						Gastritis	Leaf	Decoction	Oral intake		
Eleusine indica (L.) Gaertn.	Kimbansi, Lumvumvu	*Poaceae*	a, b	0.03	0.38	Migraine	Whole plant	Maceration, pounded	Friction	Herb	Ruderal
						Chest or intercostal pain	Leaf, whole plant	Pounded	Friction		
						Splenomegaly	Root, whole plant	Pounded	Scarification		
						Prostate	Whole plant	Decoction	Oral intake		
						Asthma	Leaf	Decoction	Oral intake		
						Gastritis	Whole plant	Decoction	Oral intake		

Synoptic table of medicinal plants and their use in Kisantu and Mbanza-Ngungu terroirs
(in which a “Kisantu”, B “Mbanza-Ngungu urban area and, c “Mbanza-Ngungu rural area”).

Species	Local name	Botanic family	Recording area	UV_S	IAR	Disease	Organ	Method of preparation	Method of administration	Morpho-logy	Harvested area
Emilia coccinea (Sims) G.Don	Nkofi masa	*Asteraceae*	b	0.01	0.00	Repeated abortion or frequent delivery of stillbirths	Root	Decoction	Oral intake	Herb	Ruderal
Erigeron floribundus (Kunth) Sch.Bip.	Fumu di Kyula	*Asteraceae*	a, b	0.02	0.00	Hemorrhoids	Whole plant	Pounded	Anal route	Herb	Ruderal
						Mastitis		Decoction	Oral intake		
						Microfilariate		Decoction	Oral intake		
						Migraine		Maceration	Enema		
Eriosema glomeratum (Guill. Et Perr.) Hook.f.	*Wandu nseke*	*Fabaceae*	b	0.01	0.00	Hernia	Root	Powder	Oral intake	Herb	Savanna
Erythrina abyssinica Lam.	Kikumbu ki Nzambi	*Fabaceae*	a, c	0.02	0.00	Anemia	Leaf	Decoction	Oral intake	Tree	Savanna
						Madness	Bark	Pounded			
						Edema and yellowing of the hair	Leaf	Decoction			
Erythrococca atrovirens (Pax) Prain	Nzeke nzeke	*Euphorbiaceae*	a	0.01	0.00	Chest or intercostal pain	Leaf	Pounded	Friction	Shrub	Forest recruit
						Asthma		Powder	Oral intake		
Erythrophleum suaveolens (Guill. & Perr.) Brenan	Nkasa, epomi	*Fabaceae*	a	0.01	0.00	Matrix cancer	Root	Decoction	Fumigation	Tree	Forest gallery
						Rheumatism	Leaf	Powder	Scarification		
Euphorbia candelabrum Trémaux ex Kotschy	Songe	*Euphorbiaceae*	b	0.01	0.00	Epilepsy	Leaf	Trituration	Massage	Shrub	Ruderal
Ficus lutea Vahl	Bubu	*Moraceae*	a	0.01	0.00	Chronic scabies with itching and stink	Bark	Powder	External application	Tree	Forest gallery
Ficus thonningii Blume	Nsanda	*Moraceae*	a, c	0.01	0.00	Pertussis	Leaf	Decoction	Oral intake	Tree	Humid forest
						Headache	Root	Ointment	Friction		
Gaertnera paniculata Benth.	Kimbodia	*Rubiaceae*	b	0.01	0.00	Cough	Bark	Decoction	Oral intake	Shrub	Forest recruit
Garcinia huillensis Welw. ex Oliv.	Kisima	*Clusiaceae*	a, c	0.01	0.00	Diabetes	Leaf	Pounded	Oral intake	Shrub	Savanna
						Erectil malfunction	Fruit/semence	Decoction			
Garcinia kola Heckel	Ngadiadia	*Clusiaceae*	c	0.01	1.00	Epilepsy	Fruit/semence	Powder	Oral intake	Shrub	Field, home garden
Gardenia ternifolia Schumach. & Thonn. Subsp.	Kilemba nzau	*Rubiaceae*	a, b, c	0.02	0.40	Sexual weakness or impotence	Root	Pounded, decoction	Oral intake	Shrub	Savanna
						Hernia	Root	Decoction	Oral intake		
						Tooth decay	Fruit/semence	Infusion	Gargle		
						Chest or intercostal pain	Bark	Pounded	Oral intake		
Gnetum africanum Welw.	Mfumbu	*Gnetaceae*	a	0.01	0.00	Diabetes	Leaf	Powder	Oral intake	Liana	Forest gallery
Gossypium barbadense L.	Gusu	*Malvaceae*	a, b, c	0.05	0.10	Tooth decay	Leaf	Decoction	Oral intake	Shrub	Field, home garden
						Heaviness in the head, seems to split in the baby		Maceration	Oral intake		
						Breast care to keep it upright		Powder	Scarification		
						Lack of uvula causing the baby to cry continuously		Maceration	Oral intake		
						Chest or intercostal pain		Pounded	Friction		
						Laryngitis		Maceration	Oral intake		
						Chronic scabies with itching and stink		Powder	Friction, external application		

Synoptic table of medicinal plants and their use in Kisantu and Mbanza-Ngungu terroirs
(in which a “Kisantu”, B “Mbanza-Ngungu urban area and, c “Mbanza-Ngungu rural area”).

Species	Local name	Botanic family	Recording area	UV_S	IAR	Disease	Organ	Method of preparation	Method of administration	Morpho-logy	Harvested area
						Enuresis		Decoction	Oral intake		
						Skin rash		Maceration	Oral intake		
						Tuberculosis		Maceration	Oral intake		
Habenaria laurentii De Wild.	Mumpemba	*Orchidaceae*	c	0.01	0.00	Erectil malfunction	Leaf	Powder	Scarification	Herb	Forest gallery
Hallea stipulosa (DC.) J.-F.Leroy	Nlongo	*Rubiaceae*	a, b	0.03	0.20	Sexual weakness or impotence	Bark	Pounded	Oral intake	Tree	Swamp forest
						Hernia		Decoction, macération			
						Hemorrhoids		Pounded			
						Female infertility		Decoction			
						Mastitis		Decoction			
Harungana madagascariensis Lam. ex Poir.	Ntunu	*Hypericaceae*	a	0.02	0.00	Diarrhea	Bud	Raw state	Oral intake	Shrub	Forest fallow
						Jaundice	Bark	Decoction			
						Gonorrhea	Bark	Decoction			
						Hemorrhoids	Root	Maceration			
Heinsia crinita (Wennberg) G.Taylor	Nsiamuna, Kita-mata	*Rubiaceae*	a, b, c	0.05	0.11	Hemorrhoids	Root	Decoction	Oral intake	Shrub	Forest recruit
						Sexual weakness or impotence		Decoction			
						Premature ejaculation		Decoction			
						Madness		Pounded			
						Rheumatism		Decoction			
						Erectil malfunction		Decoction			
						Mastitis		Decoction			
						Hernia		Decoction			
						Gastritis		Decoction			
Helichrysum mechowianum Klatt	Ludimi lua mbua	*Asteraceae*	a	0.01	0.00	Circumcision	Leaf	Buckling	External application	Herb	Savanna
Hibiscus acetosella Welw. ex Hiern	Nsa, Ngayi-ngayi	*Malvaceae*	a, c	0.02	0.00	Female infertility	Root	Decoction	Oral intake	Herb	Field, home garden
						Epistaxis with sometimes sudden death	Leaf	Decoction			
						Mastitis	Leaf	Pounded			
Hymenocardia acida Tul.	Mpete, Kigéete	*Phyllanthaceae*	a, b	0.04	0.13	Sexual weakness or impotence	Bark, root	Pounded	Application on the organ, oral intake	Shrub	Savanna
						Tooth decay	Bark	Decoction	Gargle		
						Breastfeeding	Leaf	Decoction	Oral intake		
						Wound	Bark	Powder	Enema		
						Otitis	Fruit/semence	Trituration	Instillation		
						Diarrhea	Root	Decoction	Oral intake		
						Anemia	Bark	Decoction	Oral intake		
						Hernia	Root	Decoction	Oral intake		
Hyptis suaveolens (L.) Poit.	Nkama n'songo	*Lamiaceae*	b	0.01	0.00	Rheumatism	Leaf	Ointment	Massage	Herb	Ruderal
						Epilepsy		Raw state	Oral intake		
Impatiens irvingii Hook.f.	Nsa lua nsa	*Balsaminaceae*	b	0.01	0.00	Headache	Bark	Trituration	Instillation	Herb	Ruderal
Imperata cylindrica (L.) P.Beauv.	N'sonia	*Poaceae*	a, c	0.02	0.33	Asthma	Root	Powder	Oral intake	Herb	Savanna
						Sciatic neuralgia		Decoction			
						Sexual weakness or impotence		Infusion			

Synoptic table of medicinal plants and their use in Kisantu and Mbanza-Ngungu terroirs
(in which a “Kisantu”, B “Mbanza-Ngungu urban area and, c “Mbanza-Ngungu rural area”).

Species	Local name	Botanic family	Recording area	UV_S	IAR	Disease	Organ	Method of preparation	Method of administration	Morpho-logy	Harvested area
Indigofera capitata Kotschy	Nkeka za ngo	*Fabaceae*	a	0.01	0.00	Diabetes	Whole plant	Powder	Oral intake	Herb	Savanna
Jatropha curcas L.	Mpuluka kongo	*Euphorbiaceae*	a, b, c	0.03	0.20	Gastritis	Latex/sap, leaf	Maceration, decoction	Oral intake	Tree	Field, home garden
						Hemorrhoids	Leaf	Decoction	Oral intake		
						Diarrhea	Fruit/semence	Raw state	Oral intake		
						Madness	Leaf	Pounded	Oral intake		
						Prostate	Leaf	Maceration	Oral intake		
Kalaharia uncinata (Schinz) Moldenke	Nkongi Nkongi	*Lamiaceae*	a, b, c	0.02	0.25	Laryngitis	Root	Decoction	Oral intake	Tree	Savanna
						Nephritis		Pounded			
						Testicular disappearance		Decoction			
						Hernia		Decoction			
Landolphia camptoloba (K.Schum.) Pichon	Mbungu mbungu	*Apocynaceae*	a	0.01	0.00	Asthma	Leaf	Powder	Oral intake	Liana	Savanna
Landolphia owariensis P.Beauv.	Goki di kuku	*Apocynaceae*	c	0.01	0.00	Hemorrhoids	Fruit/semence	Infusion	Oral intake	Liana	Savanna
Lannea antiscorbutica (Hiern) Engl.	Nkumbi	*Anacardiaceae*	a, b	0.01	0.00	Chronic scabies with itching and stink	Leaf	Powder	Friction	Tree	Savanna
						Tooth decay	Bark	Decoction	Fumigation		
Lantana camara L.	Nsudi nsudi	*Verbenaceae*	a, c	0.01	0.50	Cough	Leaf	Pounded, decoction	Oral intake	Shrub	Ruderal
						Hemorrhoids	Leaf, fruit	Pounded	Anal route		
Lasimorpha senegalensis Schott	Kilodia	*Araceae*	b	0.01	0.00	Malnutrition and kwashiorkor	Leaf	Decoction	Oral intake	Herb	Swamp forest
Lippia multiflora Moldenke	Bulukutu	*Verbenaceae*	a, c	0.01	0.50	Epilepsy	Leaf	Powder	Oral intake	Shrub	Field, home garden
						Diabetes		Decoction			
Luffa cylindrica (L.) M.Roem.	Tsanu	*Cucurbitaceae*	b	0.01	0.00	Gonorrhea	Leaf	Maceration	Oral intake	Liana	Ruderal
Lygodium microphyllum (Cav.) R.Br.	Kizola nkata	*Lygodiaceae*	b	0.01	0.00	Amoebiasis	Leaf	Decoction	Oral intake	Liana	Humid forest
Macaranga schweinfurthii Pax	Mfumfu	*Euphorbiaceae*	b	0.01	0.00	Healing	Latex/sap	Raw state	External application	Tree	Humid forest
Macaranga spinosa Müll.Arg.	Sasa	*Euphorbiaceae*	b	0.01	0.00	Hemorrhoids	Root	Powder	Anal route	Tree	Humid forest
Undetermined	Malungula	-	b	0.01	0.00	Anemia	Leaf	Decoction	Oral intake	Herb	Savanna
Mangifera indica L.	Manga	*Anacardiaceae*	a, b, c	0.02	0.75	Hemorrhoids	Bark, Root	Infusion, decoction, pounded, ointment, maceration	Oral intake	Tree	Field, home garden
						Gonorrhea	Bark	Decoction	Oral intake		
						Hernia	Bark	Maceration	Oral intake		
Manihot esculenta Crantz	Dioko	*Euphorbiaceae*	a, c	0.05	0.00	Laryngitis	Leaf	Decoction	Oral intake	Shrub	Field, home garden
						Migraine	Stem	Powder	Friction		
						Sexual weakness or impotence	Tuber	Pounded	Oral intake		
						Headache	Stem	Powder	Friction		
						Heaviness in the head, seems to split in the baby	Leaf	Maceration	Oral intake		
						Vitiligo	Stem	Powder	Oral intake, external application		

Synoptic table of medicinal plants and their use in Kisantu and Mbanza-Ngungu terroirs
(in which a “Kisantu”, B “Mbanza-Ngungu urban area and, c “Mbanza-Ngungu rural area”).

Species	Local name	Botanic family	Recording area	UV_S	IAR	Disease	Organ	Method of preparation	Method of administration	Morpho-logy	Harvested area
						Repeated abortion or frequent delivery of stillbirths	Leaf	Maceration	Enema		
						Amoebiasis	Tuber	Pounded	Oral intake		
						Hernia	Tuber	Raw state	Oral intake		
						Conjunctivitis and cataract	Tuber	Pounded	Instillation		
Manotes expansa Sol. ex Planch.	Dila dila	*Connaraceae*	b	0.01	0.00	Anemia	Leaf	Decoction	Oral intake	Liana	Forest recruit
Maprounea africana Müll.Arg.	Kisiedi-siedi	*Euphorbiaceae*	a, b	0.02	0.00	Female infertility	Bark	Decoction	Oral intake	Shrub	Savanna
						Tooth decay	Bark	Decoction	Gargle		
						Splenomegaly	Root	Raw state	Oral intake		
Melinis minutiflora P. Beauv.	Maleka mbua	*Poaceae*	a	0.01	0.00	Hernia	Leaf	Decoction	Oral intake	Herb	Savanna
						Sciatic neuralgia		Pounded	Friction		
Milicia excelsa (Welw.) C.C. Berg	Nkamba, Kambala	*Moraceae*	a, b, c	0.02	0.00	Female infertility	Bark	Decoction	Oral intake	Tree	Forest gallery
						Rheumatism	Bark	Decoction	Friction		
						Hernia	Bark	Maceration	Oral intake		
						Mastitis	Leaf	Decoction	Oral intake		
Millettia laurentii De Wild.	Kiboto	*Fabaceae*	a	0.01	0.00	Diabetes	Bark	Decoction	Oral intake	Tree	Forest recruit
Millettia eetveldeana (Micheli) Hauman	Mbuenge	*Fabaceae*	a, b, c	0.02	0.00	Tooth decay	Root	Pounded	Application on the organ	Tree	Forest recruit
						Laryngitis		Decoction	Oral intake		
						Hemorrhoids		Maceration	Oral intake		
						Otitis		Pounded	Instillation		
Millettia versicolor Welw. ex Baker	Mbota	*Fabaceae*	a, b	0.02	0.00	Repeated abortion or frequent delivery of stillbirths	Leaf	Maceration	Enema	Tree	Forest recruit
						Hemorrhoids		Decoction	Oral intake		
						Mycosis		Maceration	External application		
						Epilepsy		Raw state	Oral intake		
Mimosa pudica L.	Kifua ngambu, Kanga nzô	*Fabaceae*	a, c	0.01	0.00	Hemorrhoids	Whole plant	Powder	Oral intake	Herb	Ruderal
						Lack of voice in children	Leaf	Infusion			
Mitracarpus hirtus (L.) DC.	Banda nzazi	*Rubiaceae*	b, c	0.02	0.00	Mycosis	Leaf	Maceration	External application	Herb	Ruderal
						Head lice		Decoction	External application		
						Zoster		Powder	External application		
						Epilepsy		Trituration	Instillation		
Momordica charantia L.	Lumbusu	*Cucurbitaceae*	a, b, c	0.05	0.18	Mastitis	Leaf, whole plant	Decoction, pounded	Oral intake	Liana	Ruderal
						Microfilariate	Leaf, whole plant	Decoction, maceration	Oral intake		
						Sciatic neuralgia	Leaf	Decoction	Oral intake		
						Chronic scabies with itching and stink	Leaf	Powder	External application		
						Headache	Leaf	Powder	Friction		
						Measles	Whole plant	Maceration	Enema		
						Hemorrhoids	Whole plant	Maceration	Anal route		
						Skin rash	Whole plant	Maceration	Oral intake		
						Tuberculosis	Whole plant	Maceration	Oral intake		
						Prolonged crying of the baby due to intestinal parasitosis	Leaf	Maceration	Enema		

Synoptic table of medicinal plants and their use in Kisantu and Mbanza-Ngungu terroirs
(in which a "Kisantu", B "Mbanza-Ngungu urban area and, c "Mbanza-Ngungu rural area").

Species	Local name	Botanic family	Recording area	UV$_S$	IAR	Disease	Organ	Method of preparation	Method of administration	Morpho-logy	Harvested area
Mondia whitei (Hook.f.) Skeels	Kimbiolongo	*Apocynaceae*	a, b, c	0.10	0.25	Ascites	Root	Decoction	Oral intake	Liana	Forest gallery
						Cough	Whole plant	Decoction			
						Elephantiasis and yellowing of the hair	Root	Decoction			
						Hemorrhoids	Root	Decoction			
						Microfilariate	Root	Decoction			
						Itchy skin rash	Root, whole plant	Maceration, pounded			
						Enuresis	Root	Decoction			
						Epistaxis with sometimes sudden death	Root	Decoction			
						Hookworm	Whole plant	Decoction			
						Erectil malfunction	Root	Decoction			
						Gastritis	Root	Decoction, maceration			
						Rheumatism	Root	Decoction			
						Nephritis	Root	Pounded			
						Anemia	Whole plant	Decoction			
						Gonorrhea	Whole plant	Decoction			
						Testicular disappearance	Whole plant	Decoction			
						Interruption of the menstruation without being pregnant	Whole plant	Pounded			
						Sexual weakness or impotence	Root	Infusion, decoction, pounded			
						Hernia	Root	Decoction			
Monodora angolensis Welw.	Mpeya, mpeve	*Annonaceae*	a, c	0.03	0.44	Hernia	Fruit/semence	Powder	Oral intake	Tree	Humid forest
						Abdominal colic	Fruit/semence	Pounded	Friction		
						Whitlow	Fruit/semence	Pounded	External application		
						Hemorrhoids	Fruit/semence	Decoction, maceration, pounded	Oral intake, anal route		
						Erectil malfunction	Fruit/semence	Decoction	Oral intake		
						Dermatosis	Fruit/semence	Powder	Oral intake		
Morinda lucida Benth.	N'siki	*Rubiaceae*	a, b, c	0.05	0.00	Intestinal parasitosis	Leaf	Decoction	Oral intake	Tree	Forest recruit
						Tooth decay	Leaf, Root		Fumigation		
						Hookworm	Leaf		Oral intake		
						Microfilariate	Leaf		Oral intake		
						Epilepsy	Leaf		Oral intake		
						Hernia	Root		Oral intake		
						Mastitis	Leaf		Oral intake		
						Measles	Leaf		Oral intake		
						Hemorrhoids	Root		Oral intake		
Morinda morindoides (Baker) Milne-Redh.	Kongo bololo	*Rubiaceae*	a, b, c	0.04	0.00	Intestinal parasitosis	Leaf	Decoction	Oral intake	Liana	Forest gallery
						Abdominal colic	Fruit/semence	Decoction	Oral intake		
						Smallpox	Leaf	Decoction	Enema		
						Gonorrhea	Leaf	Pounded	Oral intake		
						Diabetes	Leaf	Decoction	Oral intake		
						Joint pain	Leaf	Decoction	Oral intake		
						Hemorrhoids	Leaf	Decoction	Anal route		
						Malaria	Leaf	Decoction	Oral intake		

Synoptic table of medicinal plants and their use in Kisantu and Mbanza-Ngungu terroirs
(in which a “Kisantu”, B “Mbanza-Ngungu urban area and, c “Mbanza-Ngungu rural area”).

**Species**	**Local name**	**Botanic family**	**Recording area**	**UV_S**	**IAR**	**Disease**	**Organ**	**Method of preparation**	**Method of administration**	**Morpho-logy**	**Harvested area**
Moringa oleifera Lam.	Muringa	*Moringaceae*	a	0.01	0.00	Conjunctivitis and cataract	Flower	Trituration	Instillation	Tree	Field, home garden
						Diabetes	Leaf	Powder	Oral intake		
(Undetermined)	Mpeya mpeya		a	0.01	0.00	Sexual weakness or impotence	Root	Pounded	Application on the organ	Shrub	Savanna
Mucuna pruriens (L.) DC.	Mankundia	*Fabaceae*	a, c	0.02	0.33	Epilepsy	Leaf	Powder	Oral intake	Liana	Forest fallow
						Asthma	Leaf	Powder	Oral intake		
						Tuberculosis	Leaf	Maceration	Oral intake		
Musa × paradisiaca L.	Dinkondo di matiba	*Musaceae*	a, c	0.02	0.33	Rheumatism	Bark, Fruit/semence	Powder	Scarification	Shrub	Field, home garden
						Female infertility	Root	Decoction	Oral intake		
						Hemorrhoids	Fruit/semence	Pounded	Oral intake		
Musanga cecropioides R.Br. ex Tedlie	N’senga	*Urticaceae*	a, c	0.01	1.00	Laryngitis	Bark	Decoction	Oral intake	Tree	Forest recruit
Myrianthus arboreus P.Beauv.	Mantusu, Mbuba	*Urticaceae*	c	0.01	0.00	Tuberculosis	Leaf	Maceration	Oral intake	Tree	Forest recruit
Newbouldia laevis (P.Beauv.) Seem. ex Bureau	Mumpesi mpesi (di gâta)	*Bignoniaceae*	a, c	0.03	0.33	Elephantiasis and yellowing of the hair	Root	Decoction	Oral intake	Shrub	Ruderal
						Hemorrhoids	Bark, root	Decoction, pounded	Oral intake		
						Sexual weakness or impotence	Bark, root	Decoction, pounded	Oral intake		
						Tuberculosis	Bark	Decoction	Oral intake		
						Whitlow	Leaf	Pounded	External application		
Nicotiana tabacum L.	Fumu	*Solananceae*	a, c	0.01	0.50	Hemorrhoids	Leaf	Infusion, maceration	Oral intake	Shrub	Field, home garden
						Tooth decay		Paste	Application on the organ		
Undetermined	Nkelo		a	0.01	0.00	Prostate	Leaf	Maceration	Oral intake	Herb	Savanna
Nymphaea lotus L.	Longa longa	*Nymphaeaceae*	a, b, c	0.04	0.13	Chronic scabies with itching and stink	Whole plant	Powder	External application	Herb	Swamp
						Acute nervousness	Whole plant	Pounded	Oral intake		
						Itchy skin rash	Whole plant	Maceration, pounded	Oral intake		
						Epilepsy	Whole plant	Maceration	Instillation		
						Alcoholism and smoking	Whole plant	Powder	Oral intake		
						Migraine	Leaf	Maceration	Enema		
						Edema	Leaf	Maceration	Oral intake		
						Edema and yellowing of the hair	Leaf	Decoction	Oral intake		
Ochna afzelii R.Br. ex Oliv.	Kidiimbi, Ngonti	*Ochnaceae*	a	0.01	0.00	Anemia	Bark	Decoction	Oral intake	Shrub	Savanna
Ocimum basilicum L.	Mazulu	*Lamiaceae*	a, b, c	0.04	0.00	Sudden dizziness with headache and blurred vision (Zieta)	Leaf	Pounded	Oral intake	Herb	Field, home garden
						Amoebiasis		Pounded	Anal route		
						Migraine		Trituration	Friction		
						Hemorrhoids		Decoction	Anal route		
						Hookworm		Decoction	Oral intake		
						Gastritis		Decoction, maceration	Oral intake		
						Malnutrition and kwashiorkor		Decoction	Oral intake		

Synoptic table of medicinal plants and their use in Kisantu and Mbanza-Ngungu terroirs
(in which a “Kisantu”, B “Mbanza-Ngungu urban area and, c “Mbanza-Ngungu rural area”).

**Species**	**Local name**	**Botanic family**	**Recording area**	**UV_s**	**IAR**	**Disease**	**Organ**	**Method of preparation**	**Method of administration**	**Morpho-logy**	**Harvested area**
Ocimum gratissimum L.	Dinsusu-nsusu di neni	*Lamiaceae*	a, b, c	0.08	0.26	Headache	Leaf	Crushed, trituration, pounded	Friction, instillation	Herb	Field, home garden
						Cold	Leaf	Maceration	Oral intake		
						Tooth decay	Leaf	Decoction	Oral intake		
						Asthma	Leaf	Pounded	Oral intake		
						Cough	Whole plant	Pounded	Oral intake		
						Pneumonia	Leaf	Pounded	Friction		
						Sciatic neuralgia	Leaf	Pounded	Friction		
						Slight fracture	Leaf	Trituration	External application		
						Sciatic neuralgia	Leaf	Decoction	Oral intake		
						Hemorrhoids	Leaf	Decoction	Anal route		
						Migraine	Leaf	Trituration	Instillation		
						Mastitis	Leaf	Crushed	Friction		
						Diabetes	Leaf	Decoction	Oral intake		
						Back pain	Leaf	Pounded	Anal route		
						Rheumatism	Leaf	Décoction, ointment	Friction, massage		
						Otitis	Leaf	Trituration	Instillation		
Ocimum minimum L.	Dinsusu di fioti	*Lamiaceae*	a	0.01	0.50	Migraine	Leaf	Pounded	Friction	Herb	Field, home garden
						Cough		Crushed			
Oncoba spinosa Forssk.	Nsansi	*Flacourtiaceae*	a	0.01	0.00	Syphilis	Leaf	Maceration	Oral intake	Shrub	Forest gallery
Ottelia ulvifolia (Planch.) Walp.	Làadi	*Hydrocharita-ceae*	a, b, c	0.02	0.00	Rheumatism	Leaf	Decoction	Oral intake	Herb	Swamp
						Acute nervousness	Leaf, root	Pounded	Oral intake		
						Headache	Leaf	Powder	Friction		
						Epilepsy	Leaf	Maceration	Instillation		
Parinari capensis Harv.	Nsudi funi	*Chrysobalana-ceae*	b	0.01	0.00	Diabetes	Leaf	Decoction	Oral intake	Herb	Savanna
						Malaria					
Paullinia pinnata L.	Ngudi nkayi	*Sapindaceae*	b	0.01	0.00	Pneumonia	Leaf	Trituration	Friction	Liana	Forest recruit
Pentaclethra eetveldeana De Wild. & T. Durand	Kiseka	*Fabaceae*	a	0.01	0.00	Microfilariate	Leaf	Pounded	External application	Tree	Forest recruit
Pentaclethra macrophylla Benth.	N'gansi	*Fabaceae*	a	0.01	0.00	Hernia	Bark	Decoction	Oral intake	Tree	Forest recruit
Pentadiplandra brazzeana Baill.	Nkengi kiasa	*Pentadiplandraceae*	a, b, c	0.06	0.23	Hernia	Whole plant, Root	Decoction, maceration, powder	Oral intake	Shrub	Forest gallery
						Sexual weakness or impotence	Root	Pounded	Oral intake		
						Hemorrhoids	Root	Maceration	Oral intake		
						Laryngitis	Root	Decoction	Oral intake		
						Rheumatism	Leaf	Decoction	Friction		
						Parkinson	Leaf	Decoction	Oral intake		
						Sciatic neuralgia	Root	Decoction	Friction		
						Gonorrhea	Root	Decoction	Oral intake		
						Gastritis	Leaf	Decoction	Oral intake		
						Dermatosis	Root	Powder	Oral intake		
						Mastitis	Root	Pounded	Friction		
Persea americana Mill..	Divoka	*Lauraceae*	a, b	0.01	0.00	Tooth decay	Leaf	Decoction	Fumigation	Tree	Field, home garden
						Tension	Fruit/semence		Oral intake		

Synoptic table of medicinal plants and their use in Kisantu and Mbanza-Ngungu terroirs
(in which a “Kisantu”, B “Mbanza-Ngungu urban area and, c “Mbanza-Ngungu rural area”).

Species	Local name	Botanic family	Recording area	UV_S	IAR	Disease	Organ	Method of preparation	Method of administration	Morpho-logy	Harvested area
Phaseolus vulgaris L.	Madesu	*Fabaceae*	c	0.01	0.00	Diabetes	Fruit/semence	Infusion	Oral intake	Liana	Field, home garden
Phyllanthus niruri L.	Ntéeta nteta	*Phyllanthaceae*	a, b	0.01	0.00	Migraine	Leaf	Pounded	Friction	Shrub	Ruderal
						Amoebiasis		Decoction	Oral intake		
Physalis angulata L.	Bobo	*Solananceae*	b	0.01	0.00	Gastritis	Leaf	Pounded	External application	Herb	Ruderal
Piper nigrum L.	Kupidi	*Piperaceae*	a, b, c	0.03	0.43	Hernia	Fruit/semence	Powder	Oral intake	Liana	Humid forest
						Hemorrhoids	Fruit/semence	Decoction, maceration, pounded	Oral intake		
						Hookworm	Fruit/semence	Powder	Oral intake		
						Back pain	Leaf	Pounded	Friction		
						Dermatosis	Fruit/semence	Powder	Oral intake		
Piper umbellatum L.	Lumba-lumba	*Piperaceae*	a, b	0.02	0.00	Lack of uvula causing the baby to cry continuously	Leaf	Maceration	Oral intake	Herb	Forest gallery
						Laryngitis	Leaf	Maceration			
						Alcoholism and smoking	Leaf	Powder			
Plumeria alba L.	Frangipanier	*Apocynaceae*	a	0.01	0.00	Hiccup	Latex/sap	Raw state	Instillation	Shrub	Field, home garden
Polygala acicularis Oliv.	Lusambi sambi	*Polygalaceae*	a, b, c	0.02	0.00	Diabetes	Bark	Decoction	Oral intake	Herb	Savanna
						Hemorrhoids	Leaf	Decoction	Anal route		
						Madness	Whole plant	Pounded	Oral intake		
Portulaca oleracea L.	Madia ngulu	*Portulaceae*	b	0.01	0.00	Nephritis	Whole plant	Decoction	Oral intake	Herb	Ruderal
Pseudospondias microcarpa (A.Rich.) Engl.	N'yibu	*Anacardiaceae*	a, c	0.02	0.00	Enuresis	Bark	Decoction	Oral intake	Tree	Forest gallery
						Sexual weakness or impotence		Pounded			
						Female infertility		Decoction			
						Hookworm		Powder			
Psidium cattleianum Afzel. Ex Sabine	Mfulunta	*Myrtaceae*	a	0.01	0.00	Abdominal colic	Leaf	Pounded	Friction	Shrub	Savanna
						Amoebiasis			Oral intake		
Psidium guajava L.	Dipela	*Myrtaceae*	a	0.01	0.00	Hemorrhoids	Leaf	Maceration	Oral intake	Shrub	Field, home garden
Psophocarpus scandens (Endl.) Verdc.	Kikalakasa	*Fabaceae*	b	0.01	0.00	Malnutrition and kwashiorkor	Leaf	Decoction	Oral intake	Liana	Forest fallow
Psorospermum febrifugum Spach		*Plantaginaceae*	b	0.01	0.00	Yellow fever	Bark	Decoction	Oral intake	Shrub	Savanna
Pteridium aquilinum (L.) Kuhn	Misili	*Pteridaceae*	b	0.01	0.00	Gastritis	Root	Maceration	Oral intake	Herb	Savanna
Pterocarpus angolensis DC.	Sokosoko	*Fabaceae*	a, c	0.01	0.00	Diabetes	Bark	Decoction	Oral intake	Shrub	Savanna
						Mastitis		Pounded			
Quassia africana (Baill.) Baill.	Munkadi nkadi	*Simarubaceae*	b	0.01	0.00	Diabetes	Root	Maceration	Oral intake	Shrub	Forest gallery
						Malaria	Leaf	Decoction			
Raphia textilis Welw.	Mawusu	*Arecaceae*	c	0.01	0.00	Zoster	Flower	Powder	External application	Tree	Humid forest
Rauvolfia vomitoria Wennberg	Kilungu	*Apocynaceae*	a, c	0.02	0.00	Hernia	Root	Decoction	Oral intake	Shrub	Forest fallow
						Mastitis					
						Microfilariate					
Renealmia africana Benth.	Susa	*Zingiberaceae*	c	0.01	0.00	Epistaxis with sometimes sudden death	Leaf	Decoction	Oral intake	Herb	Humid forest

Synoptic table of medicinal plants and their use in Kisantu and Mbanza-Ngungu terroirs
(in which a "Kisantu", B "Mbanza-Ngungu urban area and, c "Mbanza-Ngungu rural area").

Species	Local name	Botanic family	Recording area	UV_S	IAR	Disease	Organ	Method of preparation	Method of administration	Morpho-logy	Harvested area
Rhipsalis baccifera (J.S.Muell.) Stearn	Kisadi	*Cactaceae*	b	0.01	0.00	Nephritis	Thorn	Pounded	Oral intake	Shrub	Ruderal
						Gastritis		Decoction			
Saccharum officinarum L.	Mukuku	*Poaceae*	a, b	0.02	0.00	Hemorrhoids	Stem	Decoction	Oral intake	Herb	Field, home garden
						Female infertility	Stem	Wine			
						Tension	Leaf	Decoction			
						Gastritis	Stem	Wine			
Salacia pynaertii De Wild.	Mbondi	*Celastraceae*	b	0.01	0.00	Diabetes	Leaf	Raw state	Oral intake	Liana	Forest recruit
Sansevieria bracteata Baker	Kula nioka	*Asparagaceae*	a, c	0.01	0.00	Migraine	Leaf	Pounded	Friction	Herb	Savanna
						Stomach aches	Root	Infusion	Oral intake		
Sarcocephalus latifolius (Sm.) E.A.Bruce	Kilolo (Kienga) ki nseke	*Rubiaceae*	a, b	0.03	0.17	Hernia	Root	Decoction	Oral intake	Shrub	Savanna
						Prostate					
						Back pain					
						Female infertility					
						Mastitis					
						Sexual weakness or impotence					
Sarcocephalus pobeguinii Hua ex Pobég.	Kilolo (Kienga) ki masa	*Rubiaceae*	a, b, c	0.03	0.29	Elephantiasis and yellowing of the hair	Root	Decoction	Oral intake	Tree	Swamp forest
						Microfilariate		Decoction			
						Laryngitis		Decoction			
						Nephritis		Pounded			
						Testicular disappearance		Decoction			
						Hernia		Decoction			
Schwenckia americana L.	Tumpu di nkombo	*Solananceae*	a, b, c	0.04	0.00	Rheumatism	Leaf	Decoction	Oral intake	Herb	Ruderal
						Sciatic neuralgia	Leaf	Decoction			
						Headache	Leaf	Crushed			
						Testicular disappearance	Root	Decoction			
						Gastritis	Leaf	Decoction			
						Epilepsy	Leaf	Raw state			
						Hernia	Root	Decoction			
Scleria achtenii De Wild.	Wedi wedi, welekese	*Cyperaceae*	a	0.01	0.00	Hernia	Root	Decoction	Oral intake	Herb	Swamp
Sclerocroton cornutus (Pax) Kruijt & Roebers	Kititi	*Euphorbiaceae*	a, b	0.02	0.00	Bronchitis	Leaf	Pounded	Oral intake	Shrub	Savanna
						Female infertility	Bark	Decoction			
						Madness	Leaf	Decoction			
Sclerosperma mannii H.Wendl.	Mabondo	*Araceae*	b	0.01	0.00	Migraine	Fruit/semence	Pounded	Enema	Tree	Humid forest
Scoparia dulcis L.	Kiese kiese	*Plantaginaceae*	a	0.01	0.00	Premature ejaculation	Root	Raw state	Oral intake	Herb	Ruderal
						Polio	Leaf	Powder	Scarification		
Scorodophloeus zenkeri Harms	Kiwaya	*Fabaceae*	b, c	0.01	0.00	Hemorrhoids	Bark	Maceration	Anal route	Tree	Forest gallery
						Hookworm	Root	Powder	Oral intake		
Securidaca longepedunculata Fresen	Nkama n'sunda	*Polygalaceae*	a, b, c	0.04	0.30	Hernia	Root	Powder	Oral intake	Shrub	Savanna
						Hemorrhoids	Root	Decoction	Oral intake		
						Ovarian or tubal inflammation	Leaf	Decoction	Oral intake		
						Rheumatism	Leaf, root	Decoction, ointment	Friction, massage		
						Parkinson	Root	Decoction	Oral intake		
						Polio	Leaf, root	Decoction	Oral intake		
						Dermatosis	Root	Powder	Oral intake		
						Testicular disappearance	Root	Pounded	Oral intake		

Synoptic table of medicinal plants and their use in Kisantu and Mbanza-Ngungu terroirs
(in which a “Kisantu”, B “Mbanza-Ngungu urban area and, c “Mbanza-Ngungu rural area”).

Species	Local name	Botanic family	Recording area	UV_S	IAR	Disease	Organ	Method of preparation	Method of administration	Morpho-logy	Harvested area
Senna alata (L.) Roxb.	Bwalu,	*Fabaceae*	a	0.01	0.00	Mycosis	Leaf	Trituration	External application	Shrub	Ruderal
Senna occidentalis L	Kanga nsundi	*Fabaceae*	b	0.01	0.00	Dysmenorrhea	Root	Maceration	Oral intake	Shrub	Ruderal
Sesamum indicum L.	Wangila	*Pedaliaceae*	a, b	0.01	0.00	Paralysis<br>Madness	Fruit/semence<br>Leaf	Oil<br>Decoction	Scarification<br>Oral intake	Herb	Field, home garden
Sesamum radiatum Schumach. & Thonn.	Wangila matebo	*Pedaliaceae*	a	0.01	0.00	Gastritis	Leaf	Maceration	Oral intake	Herb	Ruderal
Sida rhombifolia L.	Lumbumvu	*Malvaceae*	a, c	0.02	0.00	Enuresis<br>Childbirth pain<br>Amoebiasis	Root<br>Leaf<br>Leaf	Decoction<br>Maceration<br>Raw state	Oral intake	Herb	Ruderal
Smilax anceps Willd.	Kikalala	*Smilacaceae*	a, c	0.01	0.00	Bronchitis<br>Microfilariate	Leaf	Pounded<br>Decoction	Oral intake	Liana	Ruderal
Solanum aethiopicum L.	Kinsumba	*Solananceae*	a, b	0.01	0.00	Belly bloating ventral<br>Gastritis	Leaf	Pounded<br>Maceration	Friction, oral intake<br>Oral intake	Herb	Field, home garden
Solanum lycopersicum L.	Lumantu	*Solananceae*	a, c	0.02	0.33	Migraine<br>Hemorrhoids<br>Headache	Leaf<br>Leaf<br>Leaf	Pounded<br>Pounded, decoction<br>Decoction	Friction<br>Oral intake, anal route<br>Inhalation	Herb	Field, home garden
Solanum melongena L.	Mbolongo	*Solananceae*	b	0.01	0.00	Bronchitis	Leaf	Trituration	Oral intake	Herb	Field, home garden
Solanum macrocarpon L.	Nkeka	*Solananceae*	a	0.01	0.00	Belly bloating ventral	Leaf	Pounded	Friction, oral intake	Shrub	Field, home garden
Solanum sp	Binsukula	*Solananceae*	a	0.01	0.00	Bronchitis	Leaf	Pounded	Oral intake	Shrub	Field, home garden
Spondias cytherea Sonn.	Manga nsendi	*Anacardiaceae*	a	0.01	0.00	Tooth decay	Bark	Decoction	Gargle	Tree	Field, home garden
Spondias mombin L.	Mungyengi	*Anacardiaceae*	b	0.01	0.00	Mastitis	Leaf	Raw state	Oral intake	Tree	Field, home garden
Steganotaenia araliacea Hochst.	Kula mvumbi, mvumbi mvumbi	*Apiaceae*	a, b	0.01	1.00	Hernia	Root	Decoction	Oral intake	Shrub	Savanna
Strychnos cocculoides Baker	Kalakonki	*Loganiaceae*	a, c	0.01	0.00	Epilepsy<br>Hernia	Leaf, root<br>Root	Powder<br>Decoction	Oral intake	Shrub	Savanna
Strychnos pungens	Mabumi	*Loganiaceae*	b	0.01	1.00	Elephantiasis and yellowing of the hair	Fruit/semence, leaf	Décoction, pounded	Oral intake, Scarification	Shrub	Savanna
Synedrella nodiflora Gaertn.	Madia ma nlumba	*Asteraceae*	b	0.01	0.00	Gastritis	Leaf	Decoction	Oral intake	Herb	Ruderal
Syzygium guineense (Willd .) DC . Subsp . Macrocarpum (Engl .) F.White	Nkizu	*Myrtaceae*	b	0.01	0.00	Urinary and vaginal infection<br>Anemia	Bark	Decoction	Oral intake	Shrub	Savanna
Tapinanthus poggei (Engl.) Danser	Kinkunda nkunda	*Loranthaceae*	a, b	0.01	0.00	Joint pain<br>Alcoholism and smoking	Leaf	Decoction<br>Powder	Oral intake	Shrub	Forest recruit
Tephrosia vogelii Hook. F.	Bwalu mbaka	*Fabaceae*	b	0.01	0.00	Mycosis<br>Itchy skin rash	Leaf	Maceration<br>Pounded	External application	Shrub	Ruderal

Synoptic table of medicinal plants and their use in Kisantu and Mbanza-Ngungu terroirs
(in which a "Kisantu", B "Mbanza-Ngungu urban area and, c "Mbanza-Ngungu rural area").

Species	Local name	Botanic family	Recording area	UV_S	IAR	Disease	Organ	Method of preparation	Method of administration	Morpho-logy	Harvested area
Tetracera alnifolia Willd.	Nzyanzi	*Dileniaceae*	a	0.01	0.00	Hernia	Leaf	Decoction	Oral intake	Liana	Swamp forest
Tetradenia riparia (Hochst.) Codd	Mutuzo	*Lamiaceae*	a	0.01	0.00	Influenza	Leaf	Trituration	Instillation	Shrub	Ruderal
Tetrorchidium didymostemon (Baill.) Pax & K. Hoffm.	Nsusa	*Euphorbiaceae*	a, c	0.02	0.00	Back pain	Root	Decoction	Oral intake	Tree	Forest gallery
						Hookworm	Leaf	Powder			
						Edema and yellowing of the hair	Leaf	Decoction			
Tithonia diversifolia (Hemsl.) A. Gray	Nkadi nkadi	*Asteraceae*	a	0.01	0.00	Malaria	Leaf	Decoction	Oral intake	Shrub	Ruderal
Trema orientalis (L.) Blume	Mundia nuni	*Cannabaeae*	b	0.01	0.00	Gastritis	Leaf	Decoction	Oral intake	Shrub	Forest recruit
Trilepisium madagscariensis	Nsekenia	*Moraceae*	b	0.01	0.00	Anemia	Bark	Decoction	Oral intake	Tree	Forest gallery
Urena lobata L.	Mpungala	*Malvaceae*	a	0.01	0.00	Diabetes	Bark	Decoction	Oral intake	Herb	Ruderal
Vernonia amygdalina Delile	Mundudi-ndudi	*Asteraceae*	a, b	0.01	0.00	Hookworm	Leaf	Decoction	Oral intake	Shrub	Savanna
						Poisoning		Pounded			
Vigna subterranea (L.) Verdc.	Nguba zi nsamba	*Fabaceae*	b	0.01	0.00	Gastritis	Fruit/semence	Maceration	Oral intake	Herb	Field, home garden
Vigna unguiculata (L.) Walp.	Madesu ma Mbuengi	*Fabaceae*	a	0.02	0.00	Chest or intercostal pain	Fruit/semence	Pounded	Scarification	Herb	Field, home garden
						Splenomegaly	Fruit/semence	Pounded	Scarification		
						Laryngitis	Fruit/semence	Decoction	Oral intake		
						Vitiligo	Leaf	Powder	Oral intake, external application		
Vitex doniana Sweet	Fiolongo	*Lamiaceae*	a	0.01	0.00	Tension	Bark	Pounded	Oral intake	Tree	Swamp forest
Vitex ferruginea Schumach. & Thonn.	Ozu	*Lamiaceae*	a	0.01	0.00	Wound	Bark	Powder	Enema	Shrub	Savanna
						Hernia		Infusion	Anal route		
Vitex madiensis Oliv.	Kifilu	*Lamiaceae*	a, b, c	0.02	0.25	Hemorrhoids	Leaf, root	Maceration, decoction	Oral intake, anal route	Shrub	Savanna
						Hookworm	Leaf	Decoction	Oral intake		
						Tension	Leaf	Decoction	Oral intake		
						Cervical lymphadenopathy	Root	Infusion	Oral intake		
Xylopia aethiopica (Dunal) A. Rich.	Nkuya nkuya	*Annonaceae*	a, b, c	0.03	0.17	Hernia	Bark, leaf	Maceration, powder	Oral intake	Tree	Swamp forest
						Ascites	Bark	Decoction			
						Hemorrhoids	Bark	Decoction			
						Sexual weakness or impotence	Fruit/semence	Decoction			
						Diabetes	Bark	Decoction			
						Polio	Bark	Decoction			
Zea mays L.	Masangu	*Poaceae*	a	0.03	0.00	Headache	Ears	Powder	Friction	Herb	Field, home garden
						Prostate	Beard, leaf	Decoction, maceration	Oral intake		
						Hemorrhoids	Leaf	Decoction	Oral intake		
						Laryngitis	Fruit/semence	Decoction	Oral intake		

Synoptic table of medicinal plants and their use in Kisantu and Mbanza-Ngungu terroirs
(in which a “Kisantu”, B “Mbanza-Ngungu urban area and, c “Mbanza-Ngungu rural area”).

**Species**	**Local name**	**Botanic family**	**Recording area**	**UV$_S$**	**IAR**	**Disease**	**Organ**	**Method of preparation**	**Method of administration**	**Morpho-logy**	**Harvested area**
Zingiber officinale Roscoe.	Tangawisi	*Zingiberaceae*	a, b, c	0.04	0.53	Hemorrhoids	Rhizome	Decoction, infusion, maceration, pounded	Oral intake, anal route	Herb	Field, home garden
						Cough	Rhizome	Decoction	Oral intake		
						Sexual weakness or impotence	Rhizome	Decoction	Oral intake		
						Premature ejaculation	Rhizome	Infusion, pounded	Oral intake		
						Gonorrhea	Rhizome	Decoction, maceration	Oral intake		
						Intestinal parasitosis	Rhizome	Decoction	Oral intake		
						Hookworm	Rhizome	Decoction	Oral intake		
						Rheumatism	Rhizome	Ointment	Massage		
